# Controlled inhibition of methyltransferases using photoswitchable peptidomimetics: towards an epigenetic regulation of leukemia†
†Electronic supplementary information (ESI) available. See DOI: 10.1039/c7sc00137a



**DOI:** 10.1039/c7sc00137a

**Published:** 2017-04-27

**Authors:** Lea Albert, Jing Xu, Ruiwei Wan, Vasundara Srinivasan, Yali Dou, Olalla Vázquez

**Affiliations:** a Fachbereich Chemie , Philipps-Universität Marburg , Hans-Meerwein-Strasse 4 , 35043 Marburg , Germany . Email: olalla.vazquez@staff.uni-marburg.de; b Department of Pathology , University of Michigan , Ann Arbor , Michigan 48109 , USA; c LOEWE Zentrum für Synthetische Mikrobiologie SynMikro , Philipps-Universität Marburg , Hans-Meerwein-Strasse 4 , 35043 Marburg , Germany

## Abstract

We describe a cell-permeable photoswitchable probe capable of modulating epigenetic cellular states by disruption of an essential protein–protein interaction within the MLL1 methyltransferase core complex. Our azobenzene-containing peptides selectively block the WDR5-MLL1 interaction by binding to WDR5 with high affinity (*K*_i_ = 1.25 nM). We determined the co-crystal structure of this photoswitchable peptiomimetic with WDR5 to understand the interaction at the atomic level. Importantly, the photoswitchable *trans* and *cis* conformers of the probe display a clear difference in their inhibition of MLL1. We further demonstrate that the designed photo-controllable azo-peptidomimetics affect the transcription of the MLL1-target gene Deptor, which regulates hematopoiesis and leukemogenesis, and inhibit the growth of leukemia cells. This strategy demonstrates the potential of photopharmacological inhibition of methyltransferase protein–protein interactions as a novel method for external epigenetic control, providing a new toolbox for controlling epigenetic states.

## Abstract 2

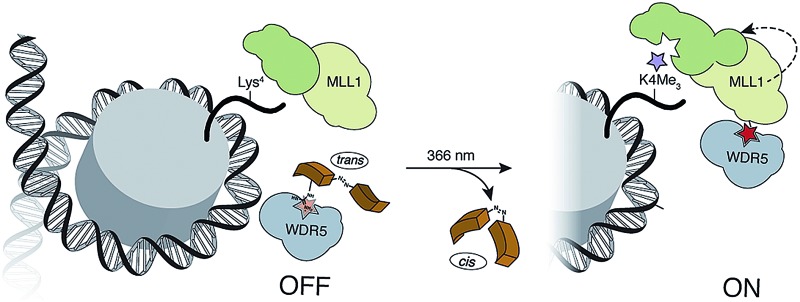
Shine light on epigenetics: we describe how photoswitchable peptidomimetics modulate the activity of the MLLl enzyme affecting epigenetic states.

## 

Epigenetics, the study of variations in gene expression unrelated to changes in the DNA sequence, is one of the most promising fields in biomedical research, since genetics alone cannot explain human variation and disease.^1^ Combinatorial post-translational modifications (PTMs) on histones, often referred to as the histone language,^2–6^ directly regulate the structure of chromatin and affect transcriptional activity by recruiting a large variety of proteins through protein–protein interactions (PPIs). One of the best-characterized histone PTMs is the specific methylation at lysine 4 of histone 3 (H3K4) by the mixed-lineage leukemia (MLL) enzymes. H3K4 trimethylation (H3K4me3) is commonly found at the promoter and enhancer regions of actively transcribed genes.^7^ Among different MLLs, MLL1 is essential for hematopoiesis^8^ and neurogenesis during embryonic development.^9,10^ It is also a promising therapeutic target; MLL1 deregulation has been linked to a subset of acute leukemia and solid tumors.^11–14^ In MLL1-rearranged leukemia cells, balanced chromosomal translocations lead to generation of MLL1 fusion proteins that include MLL1 N-terminal and C-terminal domains from several transcriptional elongation factors (AF4, AF9, ENL, and ELL).^15,16^ The C-terminus of MLL1 contains the catalytic SET domain, which is regulated by PPIs within a conserved multi-component complex.^17^ It has been shown that binding of WD40-repeat protein 5 (WDR5) to arginine 3765 (R3765) of MLL1 is crucial to enzymatic activity.^17^ Both wild type and fusion MLL1 coexist in leukemia cells and contribute to the leukemic transcription program. Despite important recent advances, the exact function of MLL1 in leukemogenesis remains unclear, highlighting the importance of developing probes for MLL1.

In recent years, there have been many advances in the development of photoresponsive probes for biological intervention that open new avenues for biological and medicinal discoveries. These technologies range from classical caged compounds to the more recent optogenetic approaches. Optogenetics,^18–21^ the use of genetically encoded photoreceptors, has shown an unprecedented potential for controlling cellular behaviour in living tissues, although in some cases its application requires complex and time-consuming genetic modifications of the proteins under study, and in some instances a simpler approach with off-the-shelf reagents might be desirable. Photopharmacology^22–25^ overcomes these limitations by taking full advantage of the small-molecule photoswitches, providing excellent delivery properties and spatio-temporal resolution with affordable probes. Epigenetic regulation could also be manipulated through the development of specific epigenetic photoswitches, as exemplified by direct targeting of the histone-deacetylase enzyme (HDAC).^26,27^ However, to our knowledge, photo-controllable probes for histone methyltransferases have not yet been reported.

Herein, we report the design and synthesis of photo-responsive probes based on azobenzene-containing peptides capable of controlling the activity of MLL1 in a reversible manner. These photo-controllable peptidomimetics target the key PPI of the MLL1 core complex: WDR5-MLL1 (Fig. 1). Furthermore, we demonstrate the potential of this reversible approach without permanent knockout of the protein and increase in scope the available chemical optoepigenetic toolbox for analysing chromatin regulation.

**Fig. 1:**
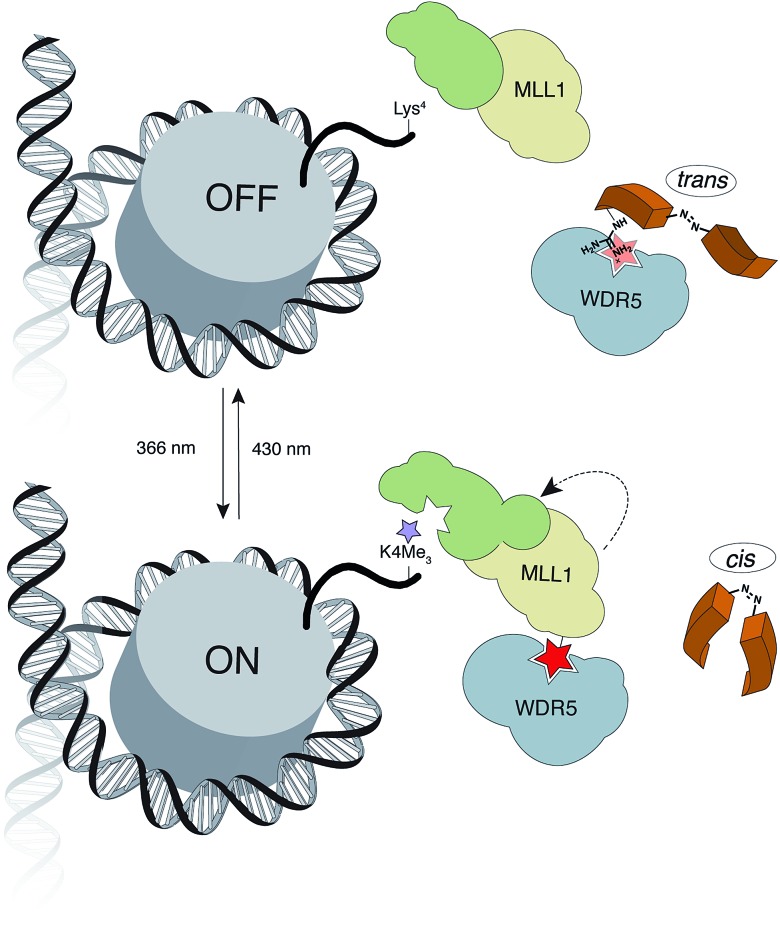
Outline of the indirect strategy for MLL1 activity control through photoswitchable inhibitors of the MLL1-WDR5 interaction.

The design of our approach is based on the recent identification of two truncations of the WDR5-INteracting peptide (WIN sequence: Ac-GS*ARA*EVHLRKS-NH_2_ (**1**)): Ac-*ARA*EVHLRKS-NH_2_ (**2**) and NH_2_-S*ARA*EVHLRKS-NH_2_ (**3**), which interact with higher affinity than the parent WIN (*K*_i_ = 160 nM, *K*_i_ = 3 nM and *K*_i_ = 20 nM for **1**, **2** and **3** respectively).^28^ The –*ARA*– sequence was identified as the indispensable motif for WDR5 recognition. Taking these promising peptide inhibitors of MLL1 as a starting point, we hypothesized that a conformational switch could reversibly modulate their affinity. Considering that the high affinity of peptide **2** could hamper the photoswitch by effectively trapping the active conformation in a stable complex with MLL1, we focused our synthetic efforts in using peptide **3** as a reference for the synthesis of our novel photoswitchable inhibitors.

As a molecular transducer we selected azobenzenes, owing to the large amount of information available on their photoresponsive properties.^29–35^ Specifically, as initial proof-of concept, we chose the 4-[(4′-aminomethyl)phenylazo]benzoic acid (AMPB)^36^ due to its synthetic simplicity, direct incorporation into the peptide backbone^37–39^ and adequate spectroscopic properties (*cis* → *trans* irradiation at 430 nm; *trans* → *cis* irradiation at 366 nm). The fact that the WIN peptide is intrinsically disordered discouraged the rational design of a photo-responsive peptide and instead suggested a systematic amino acid scan approach for the incorporation of the photoswitch. The set of peptides was prepared following the standard Fmoc-solid phase methodology and the synthesis of AMPB was carried out following literature procedures.^40^ The incorporation of this unnatural amino acid required optimization of the final TFA cleavage conditions to avoid side reactions with the azobenzene (see ESI†). All of the peptides displayed fast and reversible photoisomerization (Fig. S23†). Conversely, thermal *cis* → *trans* relaxation was a slow process (*cis*/*trans* ratio of 65 : 35 after four days in the dark; Fig. S24†), which allowed the performing of lengthy biological assays.

Once the battery of peptides was synthesized and the reversibility of their photoisomerization demonstrated, we explored whether the two photoisomers displayed suitable differences in binding affinity for WDR5 (Fig. 2). The peptide solutions were irradiated at 366 nm to generate the *cis* isomers, while the *trans* isomers were obtained through thermal relaxation since it is preferable to produce larger fold-changes between isomers.^41,42^ We determined the binding affinities using the fluorescence polarization (FP)-based competitive binding assay.^28^ Dose–response curves provide IC_50_ values, but since these data depend upon the experimental conditions, it is advisable to convert them to inhibition constants (*K*_i_) for objective comparison (Fig. 2).^43^ We verified that the excitation/emission wavelengths of the 5-carboxyfluorescein (5-FAM) tag (485/535 nm) did not interfere with the AMPB isomerization rates by recording HPLC chromatograms of the peptides before and after the assays (Fig. S29–S58†). By comparing these chromatograms with the non-irradiated ones (Fig. S1–S20†) photo-degradative processes could also be ruled out. In the same lines, the binding affinity of parent WIN **3** to WDR5 was calculated by non-irradiation and irradiation at 366 nm and 430 nm (Fig. 2 & Table S3†). Additional control experiments with AMPB itself further corroborated the absence of quenching artefacts (Fig. S27 & S28†).

**Fig. 2:**
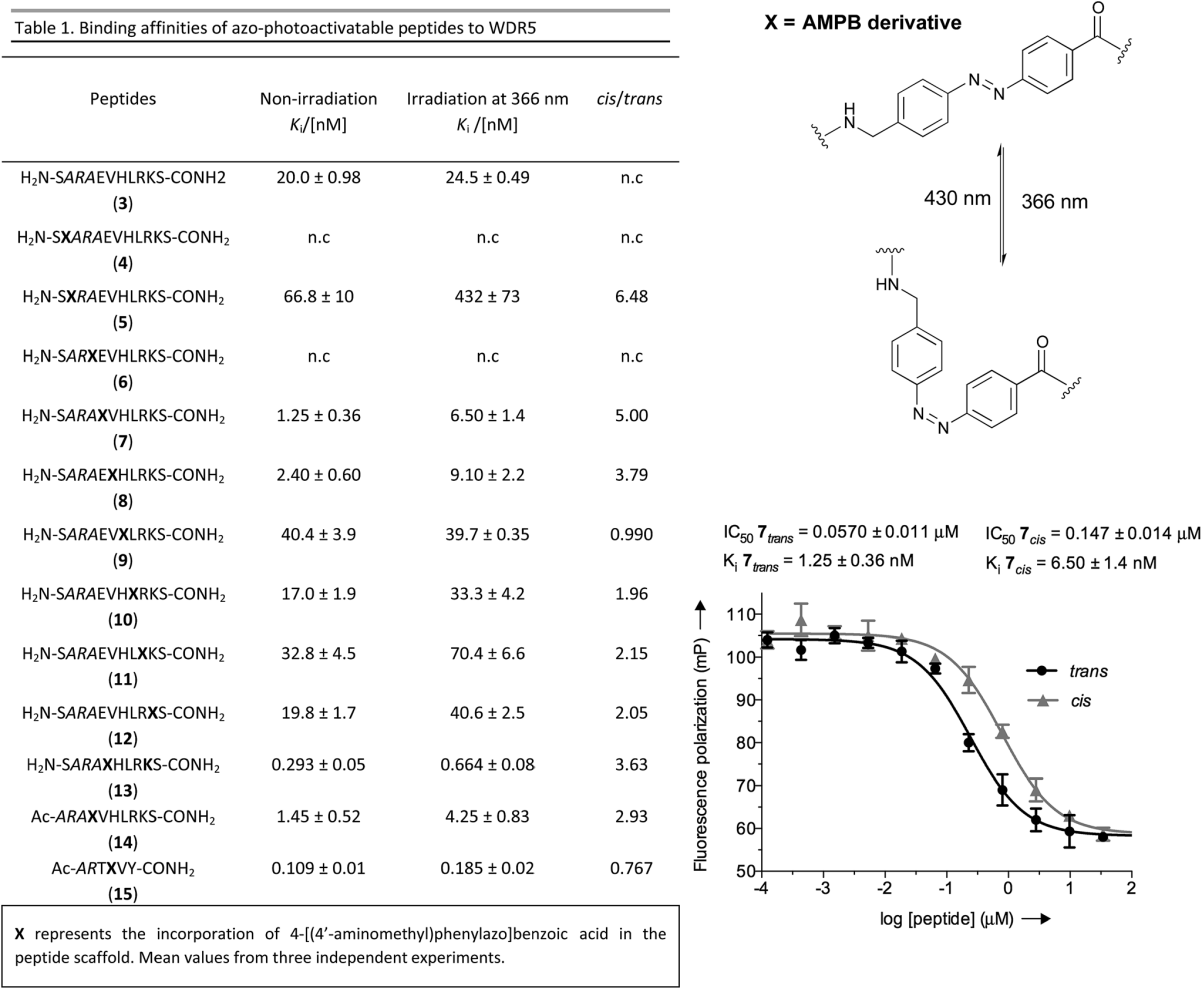
Left: table of the competitive inhibition constants (*K*_i_) to WDR5 for the azo-photoactivatable variants by FP-based competitive assays. Right: reversible photochromism of AMPB; example of the competitive binding curve for the most promising peptidomimetic **7**, IC_50_ for 5 nM of tracer.

As shown in Fig. 2, whenever there was a clear difference between both photoisomers, the *trans* isomer interacted more strongly with WDR5 than the analogous *cis* isomer. On the other hand, the effect of the AMPB switch on the WDR5-MLL1 interaction was more pronounced when it was located closer to the key arginine residue in the *ARA* minimal motif. Consequently, the N-terminal introduction of AMPB, or the replacement of both flanking alanine residues, had drastic consequences for the binding affinities, to the point that it was not possible to obtain measurable binding constants for peptidomimetics **4** and **6**. Likewise, the influence of AMPB decreased when located away from the *ARA* motif. Intriguingly, the *K*_i_ of peptidomimetics **7** and **8** decreased by an estimated factor of 10 in comparison to peptide **3**. These results agree with the best peptide inhibitors described (*K*_i_ for **2** is 3 nM). The decrease in *K*_i_ was stronger when the polar glutamic acid residue was replaced with the hydrophobic AMPB molecule. Curiously, the same modification in peptide **2**, as well as in the recently published short version Win6mer,^44^ had little influence on *K*_i_, and smaller difference between isomers (peptidomimetics **14** and **15**, respectively, Fig. 2), than the peptidomimetic **7**. Furthermore, considering the *trans* isomer of AMPB is larger than glutamic acid residue, we explored the possibility of having an increase in the difference between isomers when two residues are exchanged by AMPB (peptide **13**). However, this was not observed. Therefore, taken together these results led us to select peptidomimetic **7** for further structural studies.

To have a molecular interpretation of the increased affinity of **7** in comparison to **3**, as well as of the affinity difference between isomers, we determined the co-crystal structure of WDR5 in complex with peptidomimetic **7**, in two different states: I and II at 1.97 Å (PDB code: ; 5M23) and 2.43 Å (PDB code: ; 5M25) resolution, respectively (Fig. 3 & Table S6†). State I refers to the conditions where the *cis*/*trans* ratio of **7** was ∼5 : 95 prior to protein complexation, while in state II the *cis*/*trans* ratio of **7** was ∼80 : 20 prior to protein complexation. Furthermore, in the latter state the crystallization experiments were performed in the absence of light, and the crystal plates were always kept in the dark. In both cases, the N-terminal part of the peptide –S*ARA*– superimposes well, and the main difference between the two structures comes from the orientation of the azobenzene rings. Remarkably, in both states the *trans* isomer was the one trapped in the crystal. An interesting feature of the electron density maps of the WDR5-peptide complexes is that the C-terminal part of the peptide reveals static or molecular dynamic disorder, as reflected in the high B-values that indicate thermal displacement of atoms in crystal structures (Table S6†). The high binding affinity constants masured in solution for peptide **7** do not correlate with those seen in the crystal structures. This could be explained as an effect of the inherent dynamic nature of the peptide when bound to the protein in the crystallization buffer conditions. A similar observation has been previously described in the crystal structures of azobenzene ligands bound to streptavidin.^45^

**Fig. 3:**
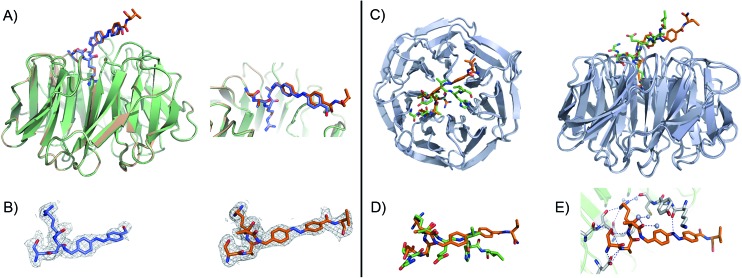
(A) Close-up view and superimposition of the state I (orange) and state II (blue) of peptidomimetic **7**; PDB code: ; 5M23 and ; 5M25, respectively. (B) A (2*F*_o_ – *F*_c_) experimental electron density map contoured at 1.0 sigma (grey) shows clear density for the peptides in both states: I (orange) and II (blue). (C) Superimposition and 90° rotation view of the WDR5 complex crystal structures with the peptidomimetic **7** (orange) and WIN peptide (green); PDB code ; 5M23 and ; 3EG6, respectively. (D) Close-up view of the two superimposed peptides: **7** and WIN. (E) Extensive hydrogen bond network of **7** with WDR5; **7** is depicted as orange sticks, interacting residues from WDR5 in white and water molecules as grey spheres.

Comparing our peptidomimetic **7** with the parent WIN (PDB code: ; 3EG6), the key interactions of **7** with the WDR5 protein in both states agree well with those in the crystal structure of the WIN peptide–WDR5 complex.^46,47^ The overlay of the two structures clearly shows that the N-terminal part of the peptide (–S*ARA*–) maintains similar interactions to the protein, as seen with the WIN peptide. However, from the glutamate onwards the orientation is different. The replacement of the solvent-exposed glutamic acid residue in the parent WIN peptide by the azobenzene ring provides additional stabilization through interaction with Lys259 and Tyr260 of MLL1 (Fig. 3E & S70†). The aromatic side chain of Tyr260 participates in van der Waals interactions with the second benzene ring of the azobenzene (CE2 and CD2 atoms of Tyr260 and CDH and CDI atoms of azobenzene, Fig. S70 and Table S7†) and Lys259 forms a hydrogen bond with the first nitrogen atom of the AMPB of peptide 7 (Fig. 3E & S70†).

The inherently nonlinear nature of biological interactions suggests that the relatively modest binding difference between the two peptidomimetic **7** isomers may be amplified and cause pronounced effects in a more realistic biological context. To test this, we studied the inhibition of MLL1 H3K4 methyltransferase activity through the previously reported *in vitro* HMT assay using the MLL1 core complex (*i.e.* MLL1, WDR5, RbBP5, and ASH2L) and the H3 20mer peptide as substrate.^17^ Gratifyingly, our assay showed that perturbation of the WDR5-MLL1 protein–protein interaction by the photoswitchable probe was sufficient to inhibit the methylation activity of MLL1. Furthermore, the difference in IC_50_ between both isomers was more than 15-fold (Fig. 4). This experiment confirmed that the photoswitchable peptidomimetic could be used to modulate MLL1 methyltrans-ferase activity. In addition, the IC_50_ obtained for the *trans* isomer was of the same order of magnitude as for MM401 (IC_50_ is 0.32 μM),^48^ which is a potent and highly specific cyclic inhibitor for MLL1, and the current published short version of Win6mer.^44^

**Fig. 4:**
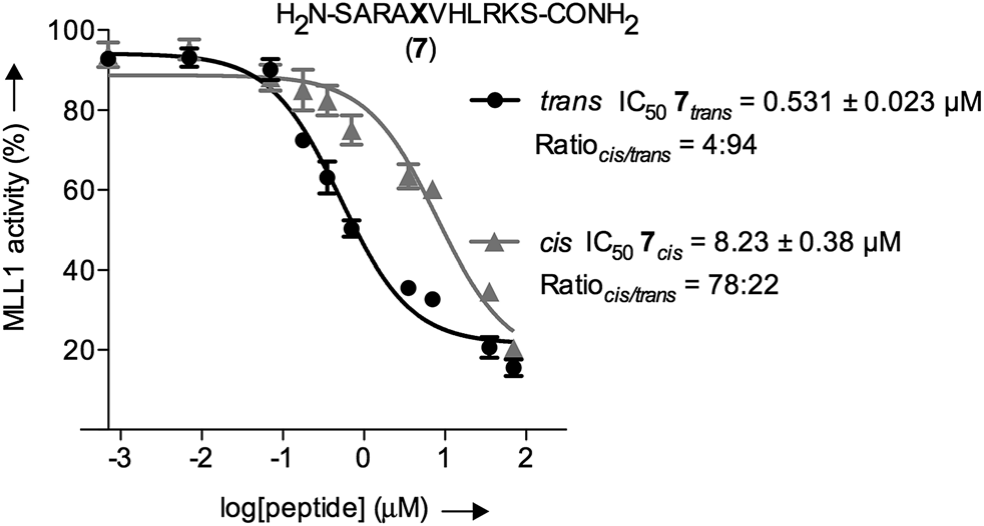
*In vitro* functional HMT assay to evaluate actual potency of our conformational trap with both peptidomimetics **7** isomers; black straight line () represents the *trans* isomer, and grey straight line (▲) represents the *cis* isomer; **X** represents AMPB. The *cis*/*trans* ratios for **7**_*trans*_ and **7**_*cis*_ were 4 : 94 and 78 : 22, respectively.

Next, we decided to investigate whether these conformationally photoswitchable peptides could be used to regulate expression of MLL1 target genes and ultimately, inhibit leukemia cell proliferation. For efficient cell internalization, two new probes with the oligo-arginine appendages: R8 (**16**) and (R-Ahx-R)_4_ (**17**),^49,50^ as well as their respective cell-penetrating peptide controls (**18** and **19**) were synthesized.

We initially studied the cellular uptake and cell viability of our compounds in murine MLL-AF9-transduced mouse bone marrow cells (Fig. 5). In line with our expectations, peptidomimetics **16** and **17** that contained peptide **7** within their sequence are the ones that inhibited the cell proliferation of leukemia. The *trans* isomers showed the highest inhibitory activity (92% for **16** and 47% for **17** at 5 μM concentration). The cell-penetrating peptide controls, as well as the unconjugated **7**, had negligible effect in comparison. Furthermore, we also prepared two analogues to **16** and **17**, which bear the parental WIN sequence **3** instead of the azo-containing peptide **7** (**20** and **21**, respectively) and compared their viability properties with our most active inhibitor (**16**_*trans*_). As shown in Fig. 6, the difference between the WIN- and the azo-containing conjugates is obvious. Thus, while in the case of the *trans* isomer of peptidomimetic **16** we already determined less than 50% of viable cells at 2.5 μM concentration, with the WIN-containing conjugates **20** and **21** we measured 87% and 96% of viable cells at 50 μM concentration, respectively.

**Fig. 5:**
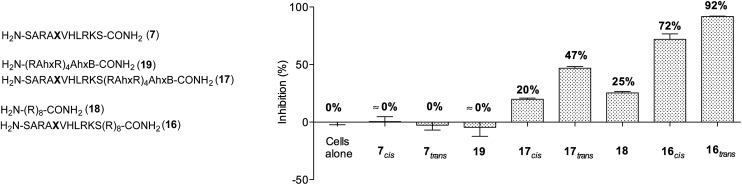
Measurements of growth inhibition of murine MLL-AF9-transduced mouse bone marrow cells after 96 h of incubation with 5 μM concentration of different peptidomimetics. Ahx, B and **X** represent aminohexanoic acid, β-alanine and AMPB, respectively.

**Fig. 6:**
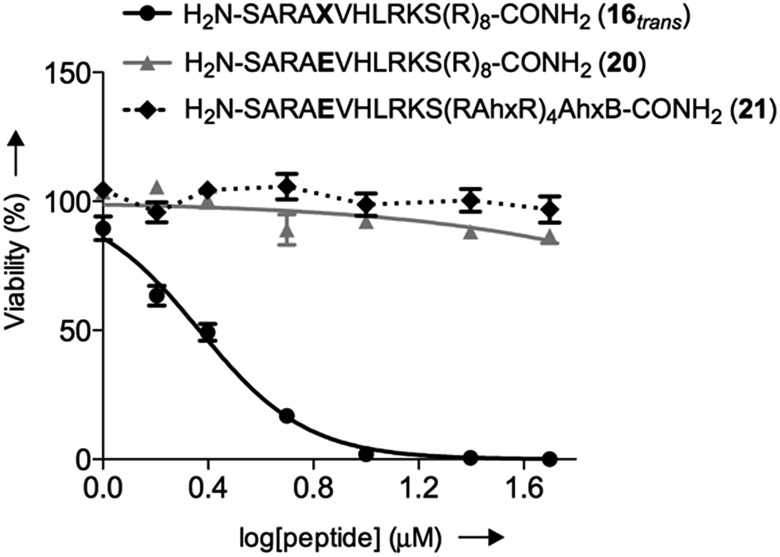
Concentration-dependent viability curves of murine MLL-AF9-transduced mouse bone marrow cells after 96 h of incubation with different compounds: black straight line () represents the *trans* isomer **16**, grey straight line (▲) represents **20** and dotted black line (♦) represents **21**; Ahx, B and **X** represent aminohexanoic acid, β-alanine and AMPB, respectively.

We then examined the potential of **16** and **17** to control externally the cellular response in proliferation assays. Thus, both isomers of both peptidomimetics were incubated at different concentrations for four days. After two days of incubation, the cells containing the *cis* isomers were irradiated at 430 nm for 90 seconds in a single isomerization cycle with a custom-made 6-well plate LED array. The possible cytotoxicity of different irradiation times, the thermal relaxation of the *cis* isomer, and its stability against glutathione for four days were also evaluated, which showed a suitable *cis*/*trans* ratio whenever the *cis* isomer was kept in the dark during the time of the experiments (see ESI†).

As a control, we also included the *cis* isomers kept in the dark for four days without any irradiation. The dose-dependent growth inhibition assays indicated that in the case of both isomers the GI_50_ was better than the compound MM401, a previously reported MLL1 inhibitor (GI_50_ for **16**_*trans*_ = 2.14 μM and GI_50_ for **17**_*trans*_ = 4.98 μM *versus* GI_50_ for MM401 = 9.76 μM; Fig. 7). More interestingly, we could observe modest, yet clear differences, up to 2-fold, in the cytotoxic behaviour of isomers in both compounds.

**Fig. 7:**
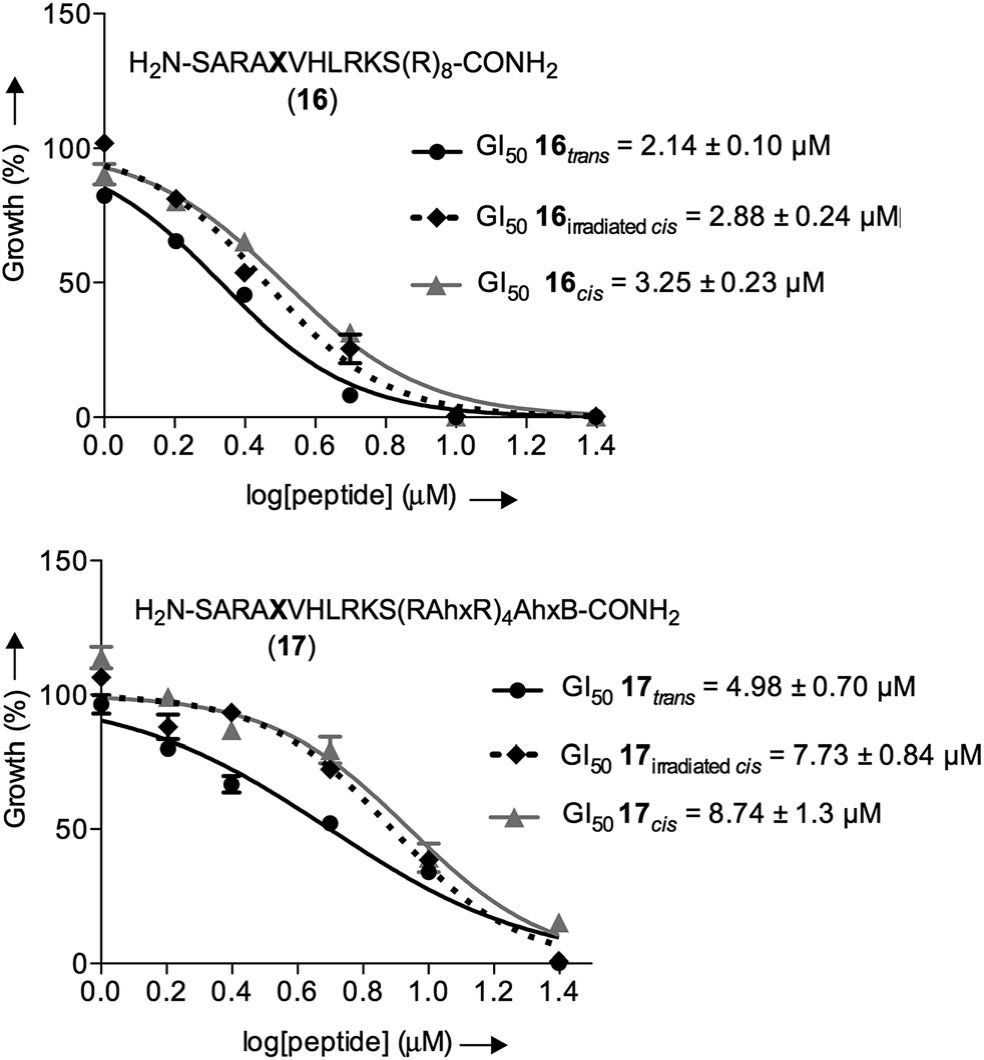
Dose-dependent growth inhibition curves for the different isomers of **16** and **17** with murine MLL-AF9-transduced mouse bone marrow cells after 96 h of incubation; black straight line () represents the *trans* isomer, dotted black line (♦) represents the irradiated *cis* isomer and grey straight line represents the *cis* isomer (▲).

These experiments confirm that our approach for targeting a key PPI with photoswitchable peptidomimetics has the potential to control enzymatic activity, leading to a distinct biological output. Our results show that it is possible to control cell proliferation through PPI photoswitches. Furthermore, our compound maintains good stability even after long periods of incubation, making potential therapeutic applications feasible.

Finally, we tested whether our probe acts as an optoepi-genetic inhibitor and controls expression of MLL1 target genes. We used the Deptor gene as a reporter, given its importance in hematopoiesis and leukemogenesis,^51,52^ as well as significant regulation by MLL1 inhibition.^53^ To this end, we examined Deptor expression by real-time PCR (RT-PCR). As expected, expression of Deptor was not affected by **7**, **18** and **19,** which also had no inhibitory effects on cell growth (Fig. 8). In contrast, treatment with peptidomimetic **16** led to significant downregulation of Deptor, comparable to MM401. Of note, a small but significant (*p* = 0.008) difference between *cis* and *trans* isomers was observed. Conjugate **17** also had a modest effect on Deptor expression, as compared to Mock (Fig. 8, *p* < 0.001). However, no statistically significant difference was found between the *trans* and *cis* isomers of **17**, probably due to lower potency of this peptidomimetic, compared to **16**. There is no statistically significant difference between *cis* isomer of **17** and the control.

**Fig. 8:**
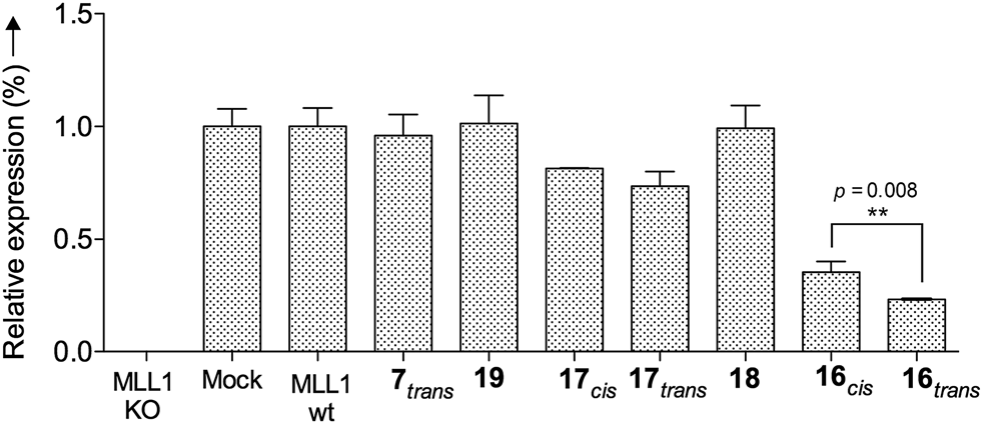
Relative Deptor mRNA expression levels in murine MLL-AF9-transduced mouse bone marrow cells after 96 h of incubation at 10 μM; they were analysed by RT-PCR and standardized to the endogenous GAPDH control. The significance is corroborated by the *p*-value.

Therefore, all these data support that our peptidomimetic **16** blocks WDR5 binding and inhibits the *in vitro* activity of MLL1 and cell proliferation of leukemia cells *via* reduction of MLL1 target gene expression.

## Conclusions

In summary, we present results on a new strategy for external epigenetic control through targeting PPIs within a multi-protein complex with peptidomimetic photoswitches, which reversibly modulate the enzymatic activity of the histone methyltransferase MLL1, and consequently affect the transcription of the Deptor gene, and the growth of leukemia cells. Light is used to trigger the conformational switch of our peptidomimetics, what is ultimately responsible of the observed difference in activity. These discoveries highlight the importance of PPIs as druggable targets^54–56^ and demonstrate the possibility of using peptide scaffolds as an efficient alternative to small-molecule inhibitors. We firmly expect that our results will encourage further developments in the use of peptidomimetics and small-molecule photoswitches in the field of epigenetics, increasing our understanding of chromatin phenomena at the molecular level.
