# Supplementary material for: Controlled inhibition of methyltransferases using photoswitchable peptidomimetics: towards an epigenetic regulation of leukemia
Source: Chem Sci. 2017 Apr 27;8(6):4612–8. doi: 10.1039/c7sc00137a (PMC5618341; doi:10.1039/c7sc00137a)
Supplement: Supplementary file 1 [file SC-008-C7SC00137A-s001.pdf]

Electronic Supplementary Material (ESI) for Chemical Science.
This journal is © The Royal Society of Chemistry 2017

Supporting Information

Controlled inhibition of methyltransferases using photoswitchable peptidomimetics: towards an epigenetic regulation of leukemia

Lea Albert[a], *Jing Xu*[b], *Ruiwei Wan*[b], *Vasundara Srinivasan*[c], *Yali Dou*[b], *Olalla Vázquez*[*a]

Contribution from: [a]Fachbereich Chemie, Philipps-Universität Marburg, Hans-Meerwein-Strasse 4, 35043 Marburg, Germany, [b]Department of Pathology, University of Michigan, Ann Arbor, Michigan 48109, United States and [c]LOEWE Zentrum für Synthetische Mikrobiologie SynMikro Philipps-Universität Marburg, Hans-Meerwein-Strasse 4, 35043 Marburg, Germany.

Table of Contents

Abbreviations I

Materials and General Procedures 1

Solid Phase Peptide Synthesis 1

Synthesis and Characterization Data of the Peptides 4

Photoisomerization of the AMPB-containing Peptides 14

UV/Vis Characterization 15

RP-HPLC Characterization 16

Expression of WDR5Δ23 18

Synthesis of the Molecular Photoswitch 19

Fluorescence Polarization-based Assays 21

Saturation Binding Experiments 22

Corroboration of Absence of Quenching Artefacts 22

Competitive Fluorescence Polarization-based Binding Assays 24

Crystal Structure of WDR5Δ23-Peptide 7 Complex 40

Evaluation of Stability against GSH-reduction of Peptides 49

In Vitro Methyl Transferase Assay with MLL1 Core Complex 50

Protein Expression 50

In Vitro Histone Methyltransferase (HMT) Assay 50

Proliferation Assay 51

Real Time Quantitative PCR Analysis of Deptor Gene 52

References 53

Abbreviations

ε	extinction coefficient
λ	wavelength
ν	frequency
A	anisotropy
A_b	anisotropy of bound tracer
A_f	anisotropy of free tracer
Ahx	aminohexanoic acid
Alloc	alkyloxycarbonyl
AMPB	(4-aminomethyl)phenyl-azobenzoic acid
Azo	azobenzene
Boc	*tert*-butylcarbonyl
c	concentration
cald.	calculated
DIC	diisopropylcarbodiimide
DIPEA	diisopropylethylamine
DMF	dimethylformamide
DMSO	dimethylsulfoxide
EC_{50}	half maximal effective concentration
EDTA	Ethylenediaminetetraacetic acid
ESI	electrospray ionization
eq.	equivalents
FAM	fluorescein
FBS	fetal bovine serum
Fmoc	fluorenylmethyloxycarbonyl chloride
FP	fluorescence polarization
HPLC	high-performance liquid chromatography
HRMS	high resolution mass spectrometry
$[I]_{50}$	concentration of free inhibitor at 50% inhibition
IC_{50}	half inhibitory concentration
IMDM	Iscove's Modified Dulbecco's Media
IPTG	isopropyl β-D-1-thiogalactopyranoside
irr.	irradiation
K_d	dissociation constant
K_i	inhibition constant
$[L]_{50}$	concentration of free labelled ligand at 50% inhibition
L_F	concentration of free ligand
L_T	total ligand concentration
mAU	milli absorbance units
MALDI	matrix-assisted laser desorption ionization
mP	milli polarization value

MPD	2-Methyl-2,4-pentanediol
NMP	*N*-methyl-2-pyrrolidon
Ni-NTA	nickel nitrilotriacetid acid
$[P]_0$	concentration of the free protein at 0% inhibition
PEG	polyethylene glycol
R_f	retention factor
R_F	concentration of free receptor
R_T	total receptor concentration
RT-PCR	real time PCR
RP	reversed phase
rpm	revolutions per minute
rt	room temperature
SDS	sodium dodecyl sulphate polyacrylamide
t_0	time zero
t_R	retention time
TFA	trifluoroacetic acid
TIS	triisopropylsilane
Tris	Tris(hydroxymethyl)aminomethane
WDR5	WD-repeat protein-5

Materials and General Procedures

All commercially purchased reagents were used without further purification as delivered from the corresponding company. The respective reagents were purchased from the following companies: DMSO biograde, NaCl biograde, Tris, 2-mercaptoethanol and DIPEA from Carl Roth (Germany), 6-aminohexanoic acid and 4-aminobenzoic acid from Alfa Aesar (Germany), Oxone from Merck (Germany), picrosulfonic acid from TCI (Germany), Fmoc-OSu from Fluorochem (UK), OxymaPure from Luxembourg Bio Technologies (Israel), DIC, glycerol and benzamidine from Acros (USA), KCl biograde, Triton and 4-aminobenzylamine from Sigma Aldrich (Germany). Fmoc-protected amino acids as well as the resin TentaGel S RAM were purchased from Iris Biotech (Germany). The rink amide MBHA resin was purchased from Fluorochem (UK). DMF was employed as peptide grade (Iris Biotech, Germany) and MeCN as HPLC grade (VWR, France). Water was purified with a Milli-Q Ultra Pure Water Purification System (TKA, Germany). All cell-based experiments were performed in compliance with University of Michigan guidelines and approved by the University Committee on the Use and Care of Animals (UCUCA).

Solid Phase Peptide Synthesis

Peptides were either synthesized manually in 2 mL polypropylen reactors with plunger and frit, (pore size 25 µm, Multi Syn Tech GmbH, Germany) or by an automated peptide synthesizer (Advanced ChemTech Apex 396). TentaGel S RAM resin (0.25 mmol/g) or a rink amide resin, where the loading of the first amino acid was determined, were used for synthesis.

Determination of the resin loading: The Fmoc group of a weighted amount of resin (~5 mg), previously loaded with the first amino acid [for 80-160 mg of rink amide resin (0.3-0.8 g/mmol): 2.00 eq. Fmoc-amino acid, 2.00 eq. OxymaPure (0.5 M in DMF), 2.00 eq. DIC were added to the resin for 45 min with a final concentration of 0.4 M of the amino acid], was deprotected by adding twice a solution of 20% piperidine in DMF for 5 min (500 uL). The filtrates were collected and the absorbance was measured at 300 nm ($\mathcal{E}_{300}$ (Fmoc) = 7800 L mol^{-1} cm^{-1})[1] on a Beckman-Coulter DU800 spectrophotometer and the loading was calculated. The remaining resin was swollen in DMF for 30 min and the non-reacted amino groups were capped following the general protocol of manual synthesis explained below.

Manual Solid Phase Protocol for Peptide Synthesis (protocol A)

The amounts of reagents of the following synthesis protocol correspond to 20-40 μmol scale. For shaking an Edmund Bühler Swip shaker was used.

Swelling: 160 mg of resin (40 μmol, loading = 0.25 mmol/g) were swelling in 1.0 mL DMF for 30 min.

Deprotection of the temporal Fmoc group: Piperidine (500 μL, 20% in DMF) was added to the resin and was shaken for 5 min. This step was repeated and the resin was filtered off and washed with DMF (5 × 1.5 mL), CH_2Cl_2 (5 × 1.5 mL), DMF (5 × 1.5 mL).

Coupling of amino acids: The Fmoc-amino acid (4.00 eq.) was dissolved in OxymaPure (0.5 M in DMF, except for the *N*-Fmoc-(4-aminomethyl)phenylazobenzoic acid (AMPB) coupling where OxymaPure was dissolved in NMP, 0.5 M, 4.00 eq.) and DIC (4.00 eq.) was added. The resulting solution was activated for 3 min and subsequently added to the resin. This suspension was shaken for 45 min. The resin was filtered off and washed with DMF (5 × 1.5 mL) and CH_2Cl_2 (5 × 1.5 mL) and DMF (5 × 1.5 mL). Monitoring of the coupling completion was confirmed by the TNBS-test[2] on few resin-beads.

Capping: A solution of lutidine/Ac_2O/DMF 6:5:89 (1.0 mL) was added to the resin and was shaken for 5 min. The resin was filtered off and washed with DMF (5 × 1.5 mL), CH_2Cl_2 (5 × 1.5 mL) and DMF (5 × 1.5 mL).

Automated Solid Phase Protocol for Peptide Synthesis (protocol B)

The amounts of reagents of the following synthesis protocol correspond to 20 μmol scale. The mixing was performed with a frequency of 300 rpm.

Swelling: 80 mg of resin (20 μmol, loading = 0.25 mmol/g) were swelling in 600 μL DMF for 30 min.

Deprotection of the temporal Fmoc group: Piperidine (550 μL, 20% in DMF) was added to the resin and was shaken for 5 min. This step was repeated and the resin was filtered off and washed with DMF (2 × 1.75 mL, and 2 × 1.00 mL with mixing for 1 min).

Coupling of amino acids: 215 μL of the respective Fmoc-amino acid (0.5 M in DMF), 215 μL of OxymaPure (0.5 M in DMF) and 121 μL of DIC (0.87 M in DMF) were added to the resin.

This suspension was mixed for 25 min. The resin was filtered and the coupling was repeated. Afterwards, the resin was washed with DMF (2 × 1.75 mL, and 2 × 1.00 mL with mixing for 1 min).

Capping: A solution of lutidine/Ac_2O/DMF 6:5:89 (550 µL) was added to the resin and was mixed for 5 min. The resin was filtered off and washed with DMF (2 × 1.75 mL, and 2 × 1.00 mL with mixing for 1 min).

After the last coupling, the resin was washed with DMF (2 × 2.2 mL, and 2 × 1.00 mL with mixing for 1 min) and CH_2Cl_2 (2 × 2.2 mL, and 3 × 1.75 mL with mixing for 1 min).

Cleavage deprotection step: This step is the same for both synthesis protocols. All the used cleavage cocktails were TFA-based, however, depending on the specific peptide they differed in the concentration of TFA, type of scavengers and reaction times. The used cleavage cocktails and reaction times are listed below.

- Cleavage cocktail A: 90% TFA, 5% DCM, 2.5% H_2O, 2.5% TIS (3 h)

- Cleavage cocktail B: 94% TFA, 5% H_2O, 1% TIS (2 h)

- Cleavage cocktail C: 95% TFA, 5% H_2O (2 h)

- Cleavage cocktail D: 94% TFA, 5% H_2O, 0.5% *m*-Cresol, 0.5% H-Cys(OMe) x HCl (2 h)

The dried resin was treated with the corresponding mixture and was shaken for the specific time. After the defined time, the resin was filtered off and the filtrate was added to dry ice-cold Et_2O (1 mL of Et_2O for 100 µL cleavage cocktail). After 10 min, the precipitated peptide was centrifuged, the supernatant was discarded and the peptide pellet was dissolved in MilliQ in order to be purified.

Purification: The probes were purified at 25 °C by preparative or semipreparative HPLC, performed on a PLC 2020 Personal Purification System (GILSON) with a preparative Nucleodur C18 HTec-column (5 µm, 250 × 16 mm; Macherey Nagel) and a flow rate of 10 mL/min or with a semipreparative C18 3001 coulumn (Phenomenex) with a flow rate of 6 mL/min. Detection of the signals was achieved with a UV detector at wavelength of 220 nm. The eluents were MilliQ H_2O (A) and MeCN (B) with addition of 0.1% TFA. The collected fractions, containing the desired peptides, were lyophilized and stored at -20 °C.

Characterization: The freeze-dried products were identified via analytical HPLC-MS on an Agilent 1200 Series HPLC-system (Agilent Technologies). If not stated differently, for all analytical HPLC runs an EC 125/2 Nucleodur 100-C18 ec column (Macherey & Nagel) using the linear gradient 5% to 40% B in 30 min with a flow rate of 0.20 mL/min was used and detection was monitored at 220 nm. Electrospray Ionization Mass Spectrometry (ESI-MS) was performed by direct injection on a *LTQ FT Finnigan* (Thermo Fischer Scientific) spectrometer. MALDI-TOF/MS spectra were recorded on an *UltraFLEX* (Bruker).

Synthesis and Characterization Data of the Peptides

Unless otherwise noted, the synthesis of the peptides was performed manually following the protocol A described above. If not noted differently, the linear gradient 5% to 40% B was used for analytical HPLC. The purity of the peptides was calculated from the integrated peak areas of the HPLC chromatograms, and is given as % for each peptide.

3: H_2N-Ser-Ala-Arg-Ala-Glu-Val-His-Leu-Arg-Lys-Ser-$CONH_2$; this peptide was synthesized according to protocol B (6 x 20 µmol, where the resins were combined after synthesis). For the final cleavage step, 551 mg of resin (loading = 0.25 mmol/g; peptide: 131.4 mg, 104.9 µmol) were treated with 14.0 mL cleavage cocktail A. After purification the product (62.7 mg, 34.4 µmol, 33%) was obtained as a white solid. t_R = 15.32 min. Purity ≥ 99%. Formula: $C_{52}H_{93}N_{21}O_{15}$. MALDI/TOF: $[M+H]^+$ cald.: 1253.4; found: 1253.4. HRMS-ESI^+ (m/z): $[M+H]^+$ cald.: 1252.7233; found: 1252.7245.

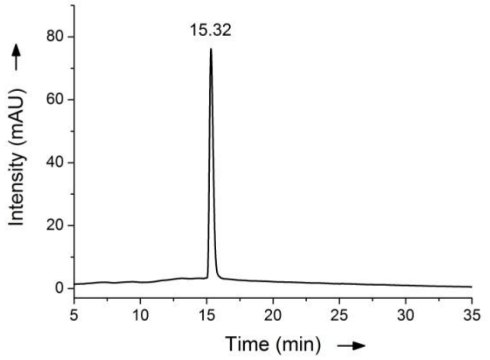

Figure S1. HPLC chromatogram of purified peptide **3**.

4: H_2N-Ser-AMPB-Ala-Arg-Ala-Glu-Val-His-Leu-Arg-Lys-Ser-$CONH_2$; for the final cleavage step, 207 mg of resin (peptide: 56.2 mg, 37.7 µmol) were treated with 10.0 mL cleavage cocktail C. After purification, the product (14.8 mg, 7.19 µmol, 19%) was obtained as an orange solid. t_R = 19.98 min (*cis*), 21.69 min (*trans*). Purity ≥ 99%. Formula: $C_{66}H_{104}N_{24}O_{16}$. MALDI/TOF: $[M+H]^+$ cald.: 1490.6; found: 1490.6. HRMS-ESI$^+$ (m/z): $[M+2H]^{2+}$ cald.: 745.4104; found: 745.4104.

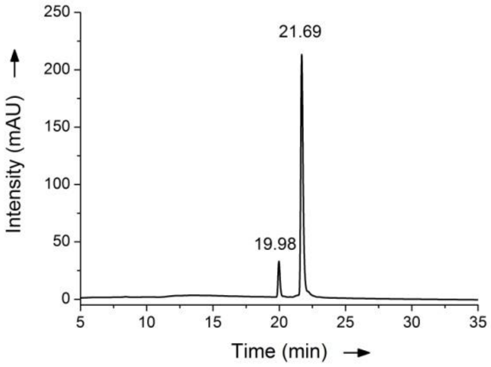

Figure S2. HPLC chromatogram of purified peptide **4**.

5: H_2N-Ser-AMPB-Arg-Ala-Glu-Val-His-Leu-Arg-Lys-Ser-$CONH_2$; for the final cleavage step, 191 mg of resin (peptide: 50.1 mg, 35.3 µmol) were treated with 10.0 mL cleavage cocktail C. After purification, the product (14.1 mg, 7.09 µmol, 20%) was obtained as an orange solid. t_R = 19.89 min (*cis*), 21.87 min (*trans*). Purity ≥ 99%. Formula: $C_{63}H_{99}N_{23}O_{15}$. MALDI/TOF: $[M+H]^+$ cald.:1419.6; found:1419.6. HRMS-ESI$^+$ (m/z): $[M+2H]^{2+}$ cald.: 709.8918; found: 709.8921.

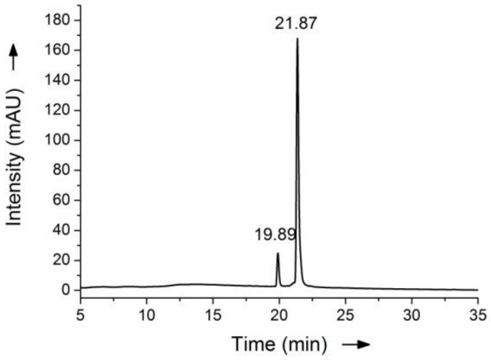

Figure S3. HPLC chromatogram of purified peptide **5**.

6: H_2N-Ser-Ala-Arg-AMPB-Glu-Val-His-Leu-Arg-Lys-Ser-$CONH_2$; for the final cleavage step, 190 mg of resin (peptide: 49.7 mg, 35.0 µmol) were treated with 10.0 mL cleavage cocktail C. After purification, the product (14.4 mg, 7.24 µmol, 21%) was obtained as an orange solid. t_R = 20.03 min (*cis*), 22.26 min (*trans*). Purity ≥ 99%. Formula: $C_{63}H_{99}N_{23}O_{15}$. MALDI/TOF: $[M+H]^+$ cald.:1419.6 found: 1419.6. HRMS-ESI$^+$ (m/z): $[M+2H]^{2+}$ cald.: 709.8918; found: 709.8921.

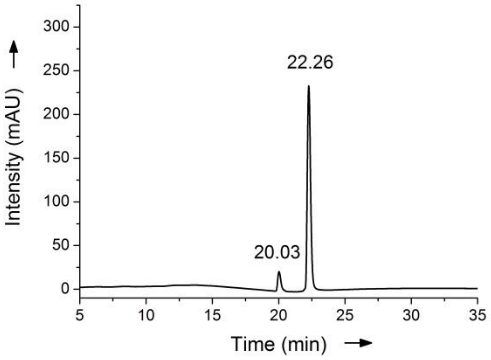

Figure S4. HPLC chromatogram of purified peptide **6**.

7: H_2N-Ser-Ala-Arg-Ala-AMPB-Val-His-Leu-Arg-Lys-Ser-$CONH_2$; for the final cleavage step, the resin (scale = 40 µmol, loading = 0.25 mmol/g) was treated with 6.00 mL cleavage cocktail C. After purification, the product (43.0 mg, 22.3 µmol, 56%) was obtained as an orange solid. t_R = 20.49 min (*cis*), 22.96 min (*trans*). Purity ≥ 99%. Formula: $C_{61}H_{97}N_{23}O_{13.}$ MALDI/TOF: $[M+H]^+$ cald.: 1361.7; found: 1361.7. HRMS-ESI$^+$ (m/z): $[M+H]^+$ cald.: 1361.7736; found: 1360.7734.

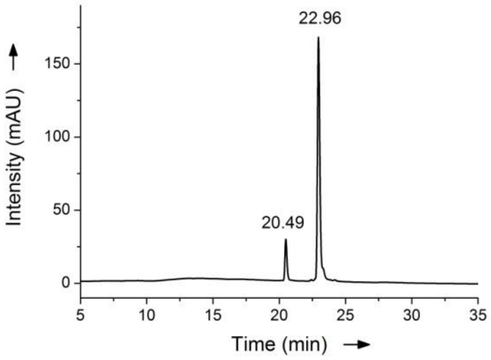

Figure S5. HPLC chromatogram of purified peptide **7**.

8: H_2N-Ser-Ala-Arg-Ala-Glu-AMPB-His-Leu-Arg-Lys-Ser-$CONH_2$; for the final cleavage step, 62.0 mg of resin (loading = 0.25 mmol/g; peptide: 15.9 mg, 11.49 µmol) were treated with 7.00 mL cleavage cocktail B. After purification, the product (1.4 mg, 1.01 µmol, 9%) was obtained as an orange solid. t_R = 19.21 min (*cis*), 21.41 min (*trans*). Purity ≥ 99%. Formula: $C_{61}H_{95}N_{23}O_{15}$. MALDI/TOF: $[M+H]^+$ cald.: 1391.7; found: 1391.7. HRMS-ESI$^+$ (m/z): $[M+H]^+$ cald.: 1390.7451; found: 1390.7491.

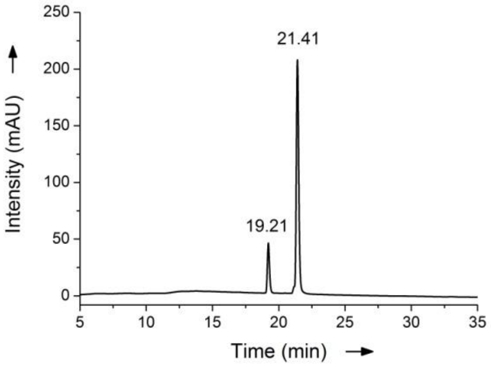

Figure S6. HPLC chromatogram of purified peptide **8**.

9: H_2N-Ser-Ala-Arg-Ala-Glu-Val-AMPB-Leu-Arg-Lys-Ser-$CONH_2$; for the final cleavage step, 109.0 mg of resin (loading = 0.25 mmol/g; peptide: 27.54 mg, 20.36 µmol) were treated with 8.00 mL cleavage cocktail D. After purification, the product (7.0 mg, 5.18 µmol, 25%) was obtained as an orange solid. t_R = 22.38 min (*cis*), 25.18 min (*trans*). Purity ≥ 99%. Formula: $C_{60}H_{97}N_{21}O_{15}$. MALDI/TOF: $[M+H]^+$ cald.: 1352.7; found: 1352.7. HRMS-ESI$^+$ (m/z): $[M+H]^+$ cald.: 1353.7573; found: 1353.7567.

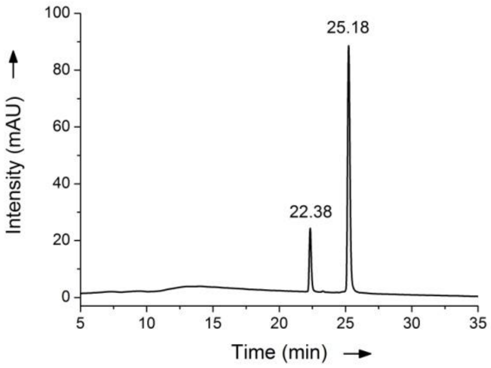

Figure S7. HPLC chromatogram of purified peptide **9**.

10: H_2N-Ser-Ala-Arg-Ala-Glu-Val-His-AMPB-Arg-Lys-Ser-$CONH_2$; for the final cleavage step, 32.0 mg of resin (loading = 0.25 mmol/g; peptide: 8.19 mg, 5.95 µmol) were treated with 3.80 mL cleavage cocktail B. After purification, the product (1.8 mg, 1.31 µmol, 22%) was obtained as an orange solid. t_R = 17.44 min (*cis*), 19.67 min (*trans*). Purity = 98%. Formula: $C_{60}H_{93}N_{23}O_{15}$. MALDI/TOF: $[M+H]^+$ cald.: 1377.7; found: 1377.7. HRMS-ESI$^+$ (m/z): $[M+H]^+$ cald.: 1377.7321; found: 1377.7337.

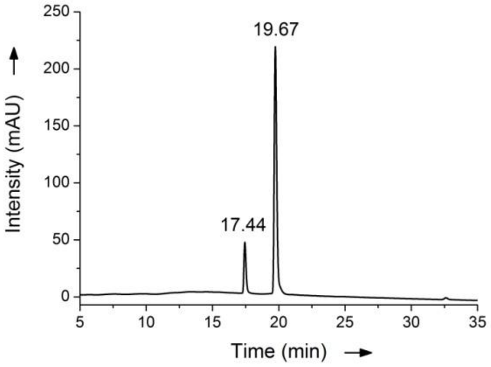

Figure S8. HPLC chromatogram of purified peptide **10**.

11: H_2N-Ser-Ala-Arg-Ala-Glu-Val-His-Leu-AMPB-Lys-Ser-$CONH_2$; for the final cleavage step, 71.5 mg of resin (rink amide, loading = 0.614 mmol/g, peptide: 32.2 mg, 24.10 µmol), were treated with 8.30 mL cleavage cocktail C. After purification, the product (9.4 mg, 5.05 µmol, 29%) was obtained as an orange solid. t_R = 22.13 min (*cis*), 23.74 min (*trans*). Purity = 99%. Formula: $C_{60}H_{92}N_{20}O_{15}$ MALDI/TOF: $[M+H]^+$ cald.: 1334.5; found: 1334.5. HRMS-ESI$^+$ (m/z): $[M+H]^+$ cald.: 1334.7152; found: 1334.7157.

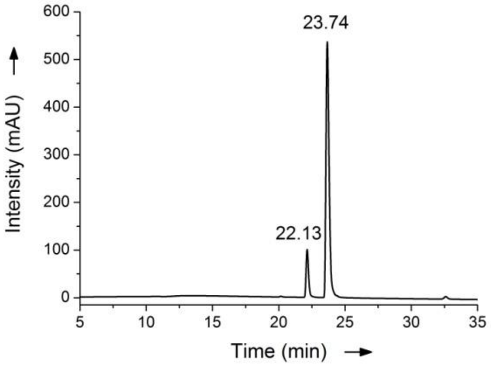

Figure S9. HPLC chromatogram of purified peptide **11**.

12: H_2N-Ser-Ala-Arg-Ala-Glu-Val-His-Leu-Arg-AMPB-Ser-$CONH_2$; for the final cleavage step, 26.0 mg of resin (rink amide, loading = 0.614 mmol/g, peptide: 11.8 mg, 8.69 µmol), were treated with 3.1 mL cleavage cocktail C. After purification, the product (7.7 mg, 5.66 µmol, 65%) was obtained as an orange solid. t_R = 21.58 min (*cis*), 23.38 min (*trans*). Purity ≥ 99%. Formula: $C_{60}H_{92}N_{22}O_{15}$. MALDI/TOF: $[M+H]^+$ cald.: 1361.5; found: 1361.5. HRMS-ESI$^+$ (m/z): $[M+H]^+$ cald.: 1362.7212; found: 1362.7289.

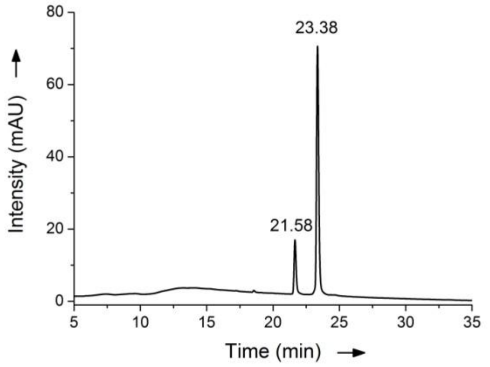

Figure S10. HPLC chromatogram of purified peptide **12**.

13: H_2N-Ser-Ala-Arg-Ala-AMPB-His-Leu-Arg-Lys-Ser-$CONH_2$; for the final cleavage step, the resin (scale = 20 µmol, loading = 0.25 mmol/g) was treated with 4.00 mL cleavage cocktail C. After purification, the product (15.7 mg, 8.57 µmol, 43%) was obtained as an orange solid. t_R = 18.57 min (*cis*), 20.64 min (*trans*). Purity = 98%. Formula: $C_{56}H_{88}N_{22}O_{12}$. MALDI/TOF: $[M+H]^+$ cald.: 1262.4; found: 1262.7. HRMS-ESI$^+$ (m/z): $[M+2H]^{2+}$ cald.: 631.3549; found: 631.3546.

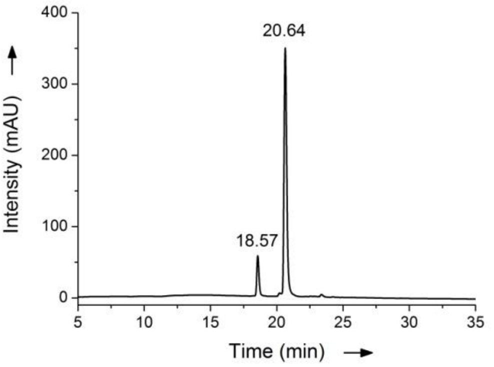

Figure S11. HPLC chromatogram of purified peptide **13**.

14: Ac-Ala-Arg-Ala-AMPB-Val-His-Leu-Arg-Lys-Ser-$CONH_2$; for the final cleavage step, 64.6 mg of resin (loading = 0.25 mmol/g; peptide: 16.0 mg, 12.0 µmol) were treated with 3.50 mL cleavage cocktail D. After purification, the product (2.70 mg, 1.52 µmol, 13%) was obtained as an orange solid. t_R = 22.79 min (*cis*), 25.21 min (*trans*) Purity ≥ 99%. Formula: $C_{60}H_{95}N_{22}O_{12}$. MALDI/TOF: $[M+H]^+$ cald.: 1316.5; found: 1316.6. HRMS-ESI$^+$ (m/z): $[M+2H]^{2+}$ cald.: 658.3784; found: 658.3793.

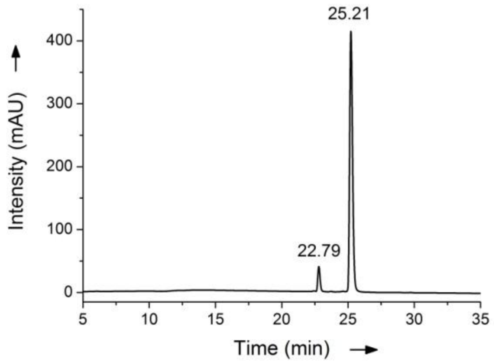

Figure S12. HPLC chromatogram of purified peptide **14**.

15: Ac-Ala-Arg-Thr-AMPB-Val-Tyr-$CONH_2$; for the final cleavage step, the resin (scale = 20 µmol, loading = 0.25 mmol/g) was treated with 4.00 mL cleavage cocktail C. After purification, the product (11.0 mg, 10.99 µmol, 55%) was obtained as an orange solid. t_R = 26.14 min (*cis*), 29.16 min (*trans*). Purity = 99%. Formula: $C_{43}H_{58}N_{12}O_9$. MALDI/TOF: $[M+H]^+$ cald.: 887.9; found: 887.9. HRMS-ESI$^+$ (m/z): $[M+H]^+$ cald.: 887.4522; found: 887.4519.

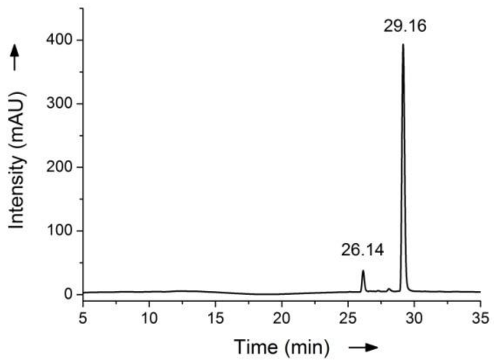

Figure S 13. HPLC chromatogram of purified peptide **15**.

16: H_2N-Ser-Ala-Arg-Ala-AMPB-Val-His-Leu-Arg-Lys-Ser-(Arg)$_8$-$CONH_2$; this peptide was synthesized manually according to protocol A, but in a higher scale (0.1 mmol). For the final cleavage step, 821 mg of resin were treated with 20 mL cleavage cocktail B. After purification, the product (86.5 mg, 0.021 mmol, 21%) was obtained as an orange solid. t_R = 20.25 min (*cis*), 22.18 min (*trans*). Purity ≥ 99%. Formula: $C_{109}H_{193}N_{55}O_{21}$. MALDI/TOF: $[M+H]^+$ cald.: 2611.3; found: 2611.3. HRMS-ESI$^+$ (m/z): $[M+5H]^{5+}$ cald.: 522.9223; found: 522.9231.

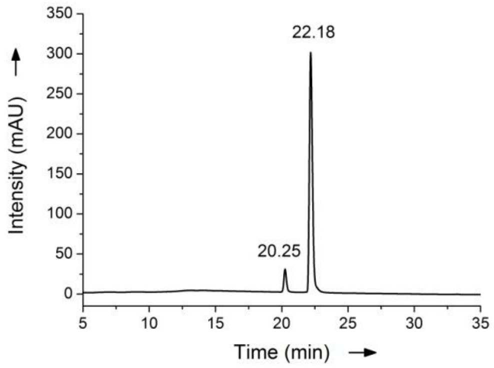

Figure S14. HPLC chromatogram of purified peptide **16**.

17: H_2N-Ser-Ala-Arg-Ala-AMPB-Val-His-Leu-Arg-Lys-Ser-(Arg-Ahx-Arg)$_4$-Ahx-βAla-$CONH_2$; this peptide was synthesized according to protocol A, but in a higher scale (0.1 mmol). For the final cleavage step, 748 mg of resin were treated with 20 mL cleavage cocktail B. After purification, the product (29.1 mg, 6.15 µmol, 6%) was obtained as an orange solid. t_R = 20.85 min (*cis*), 22.34 min (*trans*). Purity ≥ 99%. Formula: $C_{142}H_{253}N_{61}O_{27}$. MALDI/TOF: $[M+H]^+$ cald.: 3247.7; found: 3247.7. HRMS-ESI$^+$ (m/z): $[M+5H]^{5+}$ cald.: 650.2138; found: 650.2136.

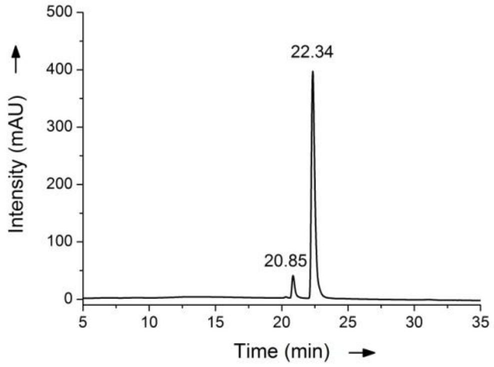

Figure S15. HPLC chromatogram of purified peptide **17**.

18: H_2N-Arg-Arg-Arg-Arg-Arg-Arg-Arg-Arg-$CONH_2$; for the final cleavage step, 216 mg of resin (peptide: 52 mg, 40 µmol) were treated with 11 mL cleavage cocktail A. After purification, the product (24.2 mg, 11.0 µmol, 27%) was obtained as a white solid. t_R = 16.33 min. Purity ≥ 99%. Formula: $C_{48}H_{99}N_{33}O_8$. MALDI/TOF: $[M+H]^+$ cald.: 1267.5; found: 1267.5. HRMS-ESI$^+$ (m/z): $[M+3H]^{3+}$ cald.: 422.9524; found: 422.9509.

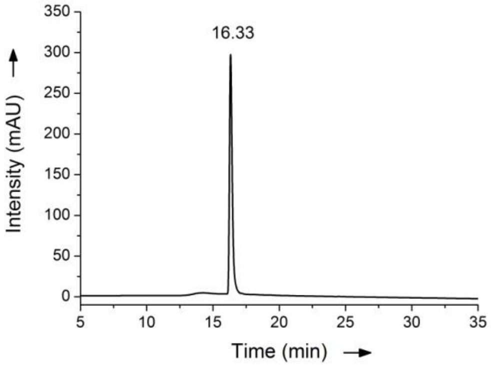

Figure S16. HPLC chromatogram of purified peptide **18** (gradient: 0-30% MeCN).

19: H_2N-(Arg-Ahx-Arg)$_4$-Ahx-βAla-$CONH_2$; for the final cleavage step, 105 mg of resin (peptide: 33.8 mg, 17.8 µmol) were treated with 5.3 mL cleavage cocktail A. After purification, the product (18.4 mg, 8.03 µmol, 45%) was obtained as a white solid. t_R = 21.98 min. Purity ≥ 99%. Formula: $C_{81}H_{159}N_{39}O_{14}$. MALDI/TOF: $[M+H]^+$ cald.: 1903.3; found: 1903.3. HRMS-ESI$^+$ (m/z): $[M+4H]^{4+}$ cald.: 476.8311; found: 476.8318.

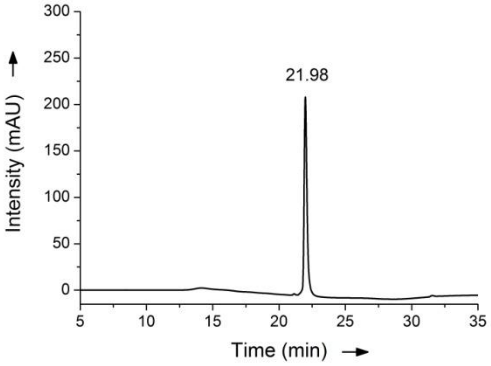

Figure S17. HPLC chromatogram of purified peptide **19** (gradient: 0-30% MeCN).

20: H_2N-Ser-Ala-Arg-Ala-Glu-Val-His-Leu-Arg-Lys-Ser-R_8-$CONH_2$; this peptide was synthesized according to protocol B (4 x 20 µmol, where the resins were combined after synthesis). For the final cleavage step, the resin (loading = 0.25 mmol/g) was treated with 16.0 mL cleavage cocktail A. After purification, the product (42.7 mg, 10.7 µmol, 13%) was obtained as a white solid. t_R = 16.88. Purity ≥ 99%. Formula: $C_{100}H_{193}N_{53}O_{23}$. MALDI/TOF: $[M+H]^+$ cald.: 2502.9; found: 2502.4. HRMS-ESI$^+$ (m/z): $[M+4H]^{4+}$ cald.: 626.3891; found: 626.3881.

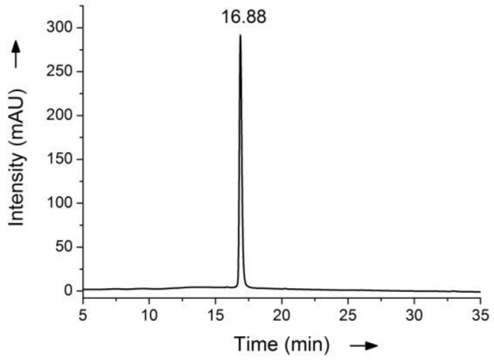

Figure S18. HPLC chromatogram of purified peptide **20**.

21: H_2N-Ser-Ala-Arg-Ala-Glu-Val-His-Leu-Arg-Lys-Ser-(Arg-Ahx-Arg)$_4$-Ahx-βAla-$CONH_2$; this peptide was synthesized according to protocol B (3 x 20 µmol, where the resins were combined after synthesis). For the final cleavage step, the resin (loading = 0.25 mmol/g) was treated with 12.0 mL cleavage cocktail A. After purification, the product (6 mg, 1.3 µmol, 2%) was obtained as a white solid. t_R = 18.39. Purity ≥ 99%. Formula: $C_{133}H_{249}N_{59}O_{29}$. MALDI/TOF: $[M+H]^+$ cald.: 3139.8; found: 3139.5. HRMS-ESI$^+$ (m/z): $[M+5H]^{5+}$ cald.: 628.6043; found: 628.6033.

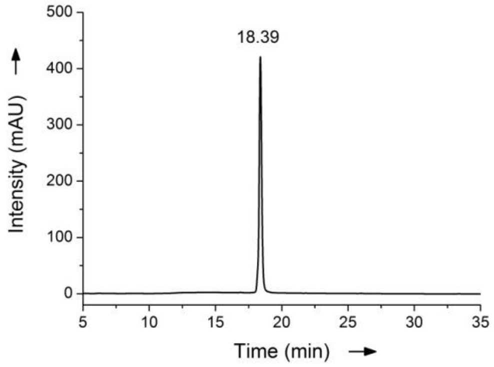

Figure S19. HPLC chromatogram of purified peptide **21**.

22: Ac-Ala-Arg-Thr-Glu-Val-His-Leu-Arg-Lys-Ser-Ahx-Ahx-Lys(FAM)-CONH$_2$; the fluorescently tagged peptide was synthesized in a 60 µmol scale using a rink amide resin (loading = 0.639 mmol/g). The *N*-terminus of the peptide was acetylated following the capping procedure explained before. The *C*-terminal lysine was introduced as Fmoc-Lys(Alloc)-OH, whose Alloc group was selectively removed in presence of standard conditions.[3,4] Pd(PPh$_3$)$_4$ (1.00 eq.), morpholine (190 eq.) and a solution of DCM with 2% water (final: 1.5 mL) were added to the resin under nitrogen atmosphere and the mixture was shaken for 5 h. The resin was filtered off and washed with DMF (3 x 2.0 mL), a solution of sodiumdiethyldithiocarbamate (29.2 mM, 2 x 2.0 mL), DMF (3 x 2.0 mL) and CH$_2$Cl$_2$ (2 x 2.0 mL). Afterwards, the 5-carboxyfluorescein (FAM) was coupled to the selectively deprotected lysine side chain according to the coupling conditions of protocol A (20 µmol). For the final cleavage, 62.0 mg of resin (peptide: 34.3 mg, 17.64 µmol) were treated with 2.5 mL cleavage cocktail A. After purification, the product (19.1 mg, 9.79 µmol, 56%) was obtained as yellow solid. t_R = 25.79 min. Purity = 99%. Formula: $C_{91}H_{136}N_{24}O_{24}$. MALDI/TOF: $[M+H]^+$ cald.: 1951.2; found: 1951.2. HRMS-ESI$^+$ (m/z): $[M+2H]^{2+}$ cald.: 975.5205; found: 975.5205.

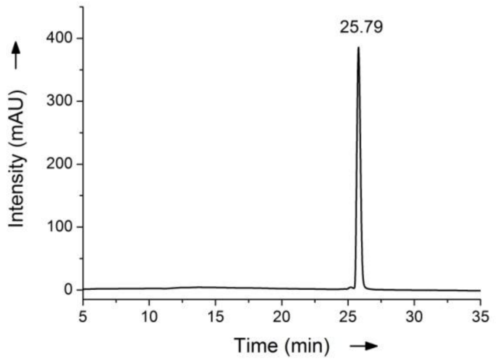

Figure S20. HPLC chromatogram of purified peptide **22**.

Photoisomerization of the AMPB-containing Peptides

A Benda (type: NU-4 KL) lamp and a Luminea spot LED E27 lamp were used for irradiation at 366 and 430 nm, respectively, in all experiments except for the proliferation assays and the RT-PCR experiments, where custom made 96- and 6-well plate LED arrays were used (LEDs: DLE-038-045 from Everlight Electronics Co. and H2A1-H435 from Roithner LaserTechnik). The photostationary states were studied by UV/Vis-spectroscopy and by analytical RP-HPLC.

UV/Vis Characterization

For the characterization via UV/Vis measurements of peptide **7**, a 500 µM solution of **7** in 0.1 M phosphate buffer (pH = 6.5) was irradiated at the respective wavelength. To obtain the *cis* isomer, the sample was irradiated at 366 nm for 1 h. For *cis* → *trans* back isomerization, the same solution was irradiated at 430 nm for 30 min afterwards. The absorbance spectra are shown in Figure S21.

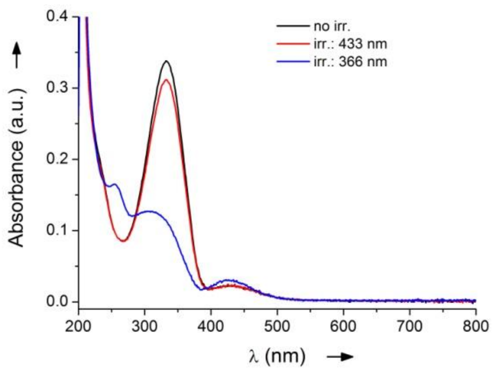

Figure S21. UV/Vis spectra of the: non-irradiated sample (black spectrum), 366 nm irradiated sample (blue spectrum) and the 430 nm irradiated sample (red spectrum).

As shown in Figure S21, the characteristic maximum of absorbance at λ_{max} = 333 nm for the π-π* transition of the *trans* isomer of the azobenzene is clearly visible in the UV/Vis-spectrum of the non-irradiated sample (black spectrum). After irradiation at 366 nm (photostationary state of the *cis* isomer), this band decreases drastically due to the loss of the *trans* isomer and the generation of the *cis* isomer, which also becomes obvious in the rise of the absorbance bands at λ_{max} = 255 nm for the π-π* and 425 nm for the n-π* transition (blue spectrum). After irradiation at 430 nm, these two bands decrease and the band at 333 nm increases again. These spectral changes are ascribed to the *cis* → *trans* back isomerization, what proves the fully reversibility of the photoisomerisation process with two isosbestic points at 287 and 394 nm. These results are consistent to the results published by MORODOER et al., who incorporated the AMPB into a different peptide.[5,6]

RP-HPLC Characterization

In the case of AMPB it is known that the *trans* isomer is the thermodynamically more stable one.[7] The maximum amount of *trans* isomer was directly obtained after purification for the whole battery of peptides. This was verified by irradiation at 430 nm (Figure S22). Both isomers could be detected by RP-HPLC, if not stated differently, using the same conditions detailed before (Section: General Materials and Methods).

Characterization of Isomerization in Phosphate Buffer (pH = 6.5)

For the isomerization, a 100 µM solution of the corresponding peptide in 0.1 M of phosphate buffer (pH = 6.5) was irradiated at the specific wavelength for different time periods. The photostationary states reached upon irradiation were determined by integrating the peak areas in the HPLC chromatograms at 287 nm (isosbestic point). All AMPB-containing peptides exhibited similar values, which are in turn also concordant with the published data.[5,8] The photoisomerization of peptide **7** is illustrated in detail.

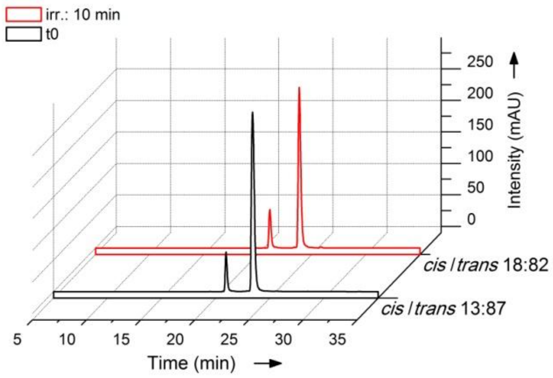

Figure S22. HPLC chromatograms of peptide **7**. After purification, the *trans* ratio is at 87% (black chromatogram). Via 10 min irradiation at 430 nm the *trans* ratio is not increasing (red chromatogram).

To assess the needed irradiation time to reach the maximum *cis* ratio, the same solution was irradiated at 366 nm for different time periods (Figure S23).

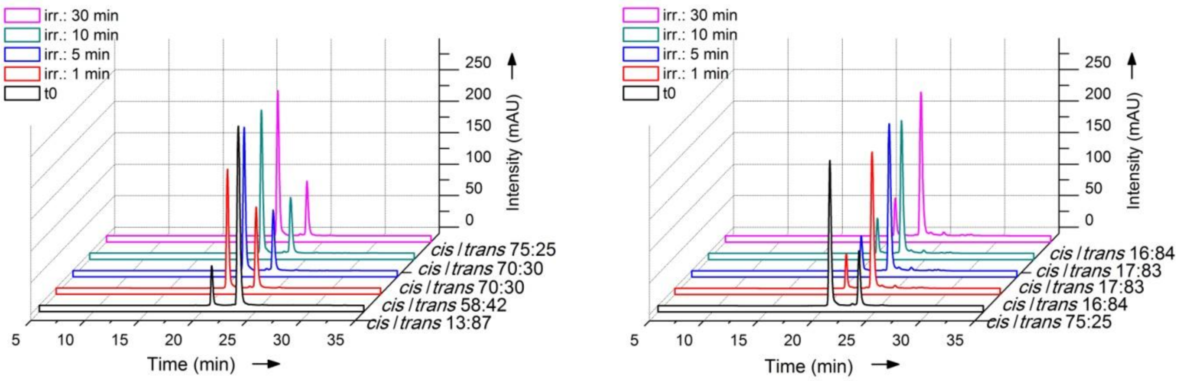

Figure S23. Left: HPLC chromatograms of peptide **7** after irradiation at 366 nm for different time periods. Right: HPLC chromatograms of peptide **7** with 75% *cis* after irradiation at 430 nm for different time periods.

Conversely, thermal *cis* → *trans* relaxation is a rather slow process.[5] A 100 µM solution of peptide **7** in 0.1 M of phosphate buffer (pH = 6.5) was irradiated at 366 nm for 30 min to obtain 76% of the *cis* isomer. Afterwards, it was stored in total darkness at rt and HPLC chromatograms, under the same conditions as before, were recorded after different time periods (Figure S24). As shown in Figure S24, after 4 days the *cis*/*trans* ratio is still at 65:35, and even after 11 days, the half-life is not yet reached.

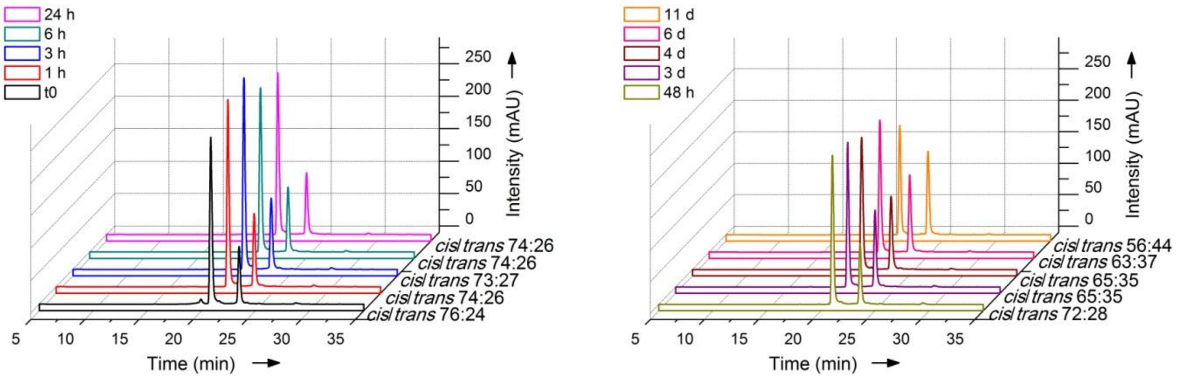

Figure S24. HPLC chromatograms of peptide **7** after the solution was irradiated at 366 nm for 30 min (black chromatogram, t0) and then stored in total darkness. HPLC chromatograms were recorded after the listed times.

Characterization of Isomerization in DMSO

The photoisomerization depends on several factors such as: solvent, concentration, intensity of the lamp etc. Therefore, the initial irradiation for the *trans* → *cis* isomerization was optimized for each peptide in the Fluorescence-Polarization based assays. The photoisomerization of peptide **7** is exemplified. For the isomerization, a 3.0 mM solution of the corresponding peptide in DMSO was irradiated at 366 nm for different time periods

(Figure S25). The irradiation to obtain the *cis* isomer was done as described above. For HPLC measurements a gradient of 5% to 30% eluent B and an Eclipse XDB-C18 column (Agilent) were used.

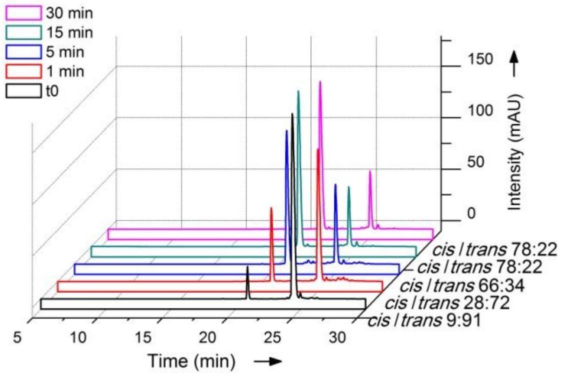

Figure S25. HPLC chromatograms after irradiation of peptide **7** in DMSO.

Expression of WDR5Δ23

The pET28a-based His-Sumo-WDR5Δ23 was expressed from the pET28-MHL vector in *E. coli* DH5α cells. Expression was performed by inoculation of 640 mL LB medium (3 times) into 2 L baffled flasks with 50 µg/mL kanamycin and 32 mL of preculture. The culture was incubated on a shaker (Multitron from Infors HT) at 37 °C and 200 rpm until an OD_{600} of 0.5-0.6. The temperature was then reduced to 25 °C and the expression was induced by adding IPTG up to a final concentration of 0.3 mM. The cells were grown overnight and harvested through centrifugation (5000 rpm, 15 min, 4 °C, Sorvall RC6 Plus from Thermo Scientific). The cell pellet from the 2 L expression culture was resuspended in 40 mL lysis buffer (400 µL PMSF (stock: 15 mg/mL), 400 µL 10% NP-40, 4 µL benzamidine (stock: 20mg/mL), 4 µL 2-mercaptoethanole in 40 mL BC500 (25 mM Tris, pH = 8.0, 500 mM NaCl, 20% glycerol)). Lysis of the cells was carried out using a sonicator Sonoplus HD 220 from Bandelin (3 x 40%, output: 4, with 2 min pulses and 3 min rest). After sonification, the suspension was pelleted by centrifugation (12000 rpm, 20 min, 4 °C). The lysate was added to Ni-NTA agarose (Quiagen, Germany) and incubated for 1 h at 4 °C. Cleavage of the His_6-tag was achieved by incubation of the resin with 3.0 mL BC150 (25 mM Tris, pH = 8.0, 150 mM NaCl, 20% glycerol) + 18.1 µL ULP-His_6. WDR5Δ23 was eluted from the resin with 3 mL of elution buffer (250 mM imidazole in BC150) and then loaded onto a Mono S 5/50 GL column (GE Healthcare) using an ÄKTA purification system (GE Healthcare). The flow rate was 1 mL/min with the Mono S buffers A (25 mM Tris, pH = 8.0, 20% glycerol) and B (25 mM

Tris, pH = 8.0, 1 M NaCl, 20% glycerol) with first an isocratic flow of 100% A for 10 min followed by a linear gradient of 0-100% B in 20 min. WDR5Δ23 eluted between 35-40% of buffer B. The fractions were checked via SDS-PAGE and pooled. The buffer was changed to the dialysis buffer (25 mM Tris, pH = 8.0, 150 mM NaCl) and the protein was concentrated up to a concentration of 275.57 µM (10.06 mg/mL). The concentrated protein was aliquoted, flash frozen in liquid nitrogen and stored at -80 °C.

Synthesis of the Molecular Photoswitch

The synthesis of the molecular photoswitch **23**, was carried out via a MILLS-condensation (Scheme S1) according to the literature quoted below. All the analytical data were consistent with the literature.

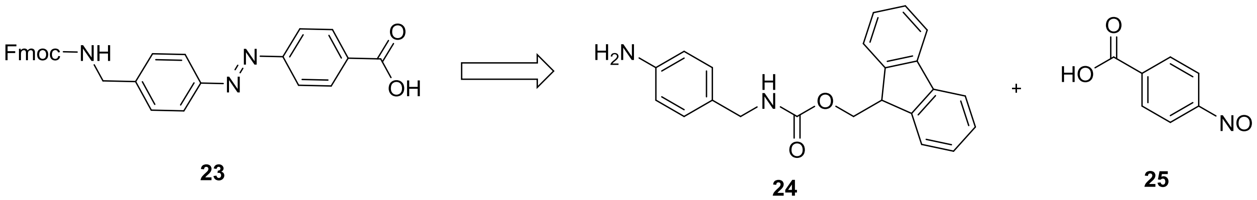

Scheme S1. Retrosynthetic approach for the *N*-Fmoc-(4-aminomethyl)phenylazobenzoic acid (**23**).

(4-Aminobenzyl)carbamic 9H-fluoren-9-yl methyl ester (24)

Following the procedure of MORODER et al.,[9] 9-fluorenylmethyl *N*-succinimidyl carbonate (101 mg, 0.30 mmol, 1.00 eq) dissolved in MeCN (0.70 mL) was added dropwise to a solution of 4-aminobenzylamine (0.03 mL, 0.30 mmol, 1.00 eq) and Et_3N (0.04 mL, 0.30 mmol, 1.00 eq) in MeCN/DMF 10:1 (0.40 mL) The solution was stirred for 1 h at rt. After filtration the precipitate was washed with methyl-*tert*-butyl-ether/trifluoroethanol 1:1 (2.0 mL). The crude product was dried and purified using flash column chromatography (*n*-Pentane/EtOAc 1:1) to yield the Fmoc-protected aminobenzylamine (48.0 mg, 0.14 mmol, 47%) as a white solid. **TLC:** R_f = 0.44 (*n*-Pentane/EtOAc 1:1) **^{1}H-NMR (300 MHz, DMSO$_{d6}$; *δ*):** 7.89 (2H, d, ^3J = 7.4, 2 x C$H_{ar,Fmoc}$), 7.69 (2H, d, ^3J = 7.4, 2 x C$H_{ar,Fmoc}$), 7.41 (2H, t, ^3J = 7.3, 2 x C$H_{ar,Fmoc}$), 7.32 (2H, t, ^3J = 7.7, 2 x C$H_{ar,Fmoc}$), 6.89 (2H, d, ^3J = 8.2, 2 x CH_{ar}), 6.49 (2H, d, ^3J = 8.2, 2 x CH_{ar}), 4.93 (s, 2H, CH_2NH), 4.31 (s, 1H, N*H*), 4.29 (s, 2H, NH_2), 4.21 (t, 1H, ^3J = 6.6, C*H*14), 3.99 (d, 2H, ^3J = 5.9, CH_2O). **^{13}C-NMR (75 MHz, DMSO$_{d6}$; *δ*):** 158.6 (*C*O_2NH), 141.9 ($C_{ar,t}$), 138.6 ($C_{ar}$$NH_2$), 135.6 (2 x $C_{ar,t}$), 131.9

(2 x $C_{ar,t}$), 127.9 (3 x $C_{ar,s}$H), 127.5 (2 x $C_{ar,s}$H), 126.9 (3 x $C_{ar,s}$H), 119.9 (2 x $C_{ar,s}$H), 113.6 (2 x $C_{ar,s}$H), 74.1 (CH_2), 60.1 (*C*H), 43.2 (CH_2). **HRMS-ESI$^+$ (m/z):** [M+Na]$^+$ calcl. for: $C_{22}H_{20}N_2O_2Na$, 367.1417; found: 367.1416.

4-Nitrosobenzoic acid (25)

Following the procedure of PRIEWISCH et al.,[10] aminobenzoic acid (3.43 g, 25.0 mmol, 1.00 eq) was suspended in DCM (40.0 mL). A solution of Oxone (30.74 g, 50.0 mmol, 2.00 eq) in H_2O (155 mL) was added and the suspension stirred for 1 h at rt. The precipitate was filtered and washed with H_2O. The crude product was freeze dried to yield 4-nitrosobenzoic acid (3.78 g, 25.0 mmol, >99.9%) as a yellow solid, which was directly used without any further purification. **TLC:** R_f = 0.39 (DCM/MeOH 9:1) **^{1}H-NMR (300 MHz, DMSO$_{d6}$; δ):** 13.41 (1H, s, O*H*), 8.27 (2H, d, ^3J = 8.6, CH_{ar}), 8.03 (2H, d, ^3J = 8.6, CH_{ar}). **^{13}C-NMR (75 MHz, DMSO$_{d6}$; δ):** 168.9 (*C*OOH), 166.9 (C_{ar}NO), 135.6 ($C_{ar,1}$), 130.4 (C_{ar}), 117.5 (C_{ar}).

N-Fmoc-(4-aminomethyl)phenylazobenzoic acid (23)

Following the procedure of PRIEWISCH et al.,[10] Fmoc-protected aminobenzylamine (1.288 g, 3.74 mmol, 1.00 eq) was added to a suspension of 4-nitrosobenzoic acid (3.78 g, 25.0 mmol, 6.68 eq) in acetic acid/DMSO 1:1 (315 mL). The suspension was stirred for 36 h at rt. After careful addition of H_2O, the precipitate was filtered off and washed with H_2O. The crude product was purified by recrystallization in acetone to yield *N*-Fmoc-(4-aminomethyl)phenylazobenzoic acid (1.39 g, 2.92 mmol, 78%) as an orange solid. **TLC:** R_f = 0.38 (DCM/MeOH 9:1) **^{1}H-NMR (300 MHz, DMSO$_{d6}$; δ):** 13.28 (s, 1H, O*H*), 8.15 (d, ^3J = 8.5, 2H, 2 x CH_{ar}), 7.93 (m, 6H, 3 x CH_{ar}), 7.71 (d, ^3J = 7.4, 2H, 2 x CH_{ar}), 7.43 (t, ^3J = 8.8, 2H, 2 x CH_{ar}), 7.34 (t, ^3J = 7.1, 2H, 2 x CH_{ar}), 4.40 (d, ^3J = 6.7, 2H, CH_2-33), 4.30 (d, ^3J = 6.0, 2H, CH_2-17), 4.24 (t, ^3J = 6.8, 1H, CH-23). **^{13}C-NMR (75 MHz, DMSO$_{d6}$; δ):** 166.6 (*C*OOH), 156.3 (*C*ONH), 154.3 (C_{ar}N), 150.9 (C_{ar}N), 144.2 (C_{ar}), 143.8 (2 x C_{ar}), 140.7 (2 x C_{ar}), 132.7 (C_{ar}), 130.55 (2 x C_{ar}H), 127.9 (2 x C_{ar}H), 127.5 (2 x C_{ar}H), 126.9 (2 x C_{ar}H), 125.1 (2 x C_{ar}H), 122.8 (2 x C_{ar}H), 122.4 (2 x C_{ar}H), 120.0 (2 x C_{ar}H), 65.3 (CH_2O), 46.8 (*C*H), 43.5 (CH_2NH). **HRMS-ESI$^-$ (m/z):** [M-H]$^-$ calcl. for: $C_{29}H_{22}N_3O_4$, 476.1616; found: 476. 1603. **IR (thin film) $\tilde{\nu}$ (cm^{-1}):** 3305 (vw), 2826 (vw), 1686 (vs), 1603 (w), 1525 (m), 1462 (w), 1448 (w), 1423 (m), 1290 (s), 1257 (s), 1141 (w), 1103 (w), 1082

(vw), 1045 (w), 1010 (w), 986 (w), 936 (w), 865 (m), 838 (w), 775 (m), 757 (m), 734 (s), 690 (m), 666 (w), 641 (w), 620 (w), 591 (w), 556 (w), 541(m), 425 (m), 400 (w), 387 (w).

(4-Aminomethyl)phenylazobenzoic acid (26)

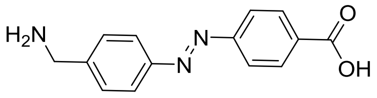

26

N-Fmoc-(4-aminomethyl)phenylazobenzoic acid (50.0 mg, 0.105 mmol, 1.00 eq) was dissolved in THF (4.12 mL) and piperidine (1.03 mL, 10.5 mmol, 100 eq) was added. The solution was stirred at rt for 2.5 h. After the reaction was completed, the solvent was evaporated under reduced pressure. The residue was washed with DCM, and the pure product (25.5 mg, 0.1 mmol, 95%) was obtained as an orange solid. **TLC:** R_f = 0.03 (DCM/MeOH 9:1) **^{1}H-NMR (300 MHz, DMSO$_{d6}$; δ):** 8.31 (s, 2H, N*H*$_2$), 8.17 (d, ^3J = 8.6, 2H, 2 x C*H*$_{ar}$), 8.01 (d, ^3J = 3.3, 2H, 2 x C*H*$_{ar}$), 7.98 (d, ^3J = 3.5, 2H, 2 x C*H*$_{ar}$), 7.71 (d, ^3J = 8.5, 2H, 2 x C*H*$_{ar}$), 4.18 (q, ^3J = 5.5, 2H, C*H*$_2$-NH$_2$). **^{13}C-NMR (75 MHz, DMSO$_{d6}$; δ):** 166.6 (*C*OOH), 154.1 (*C*$_{ar}$N), 151.7 (*C*$_{ar}$N), 137.9 (*C*$_{ar}$), 133.0 (*C*$_{ar}$), 130.6 (2 x *C*$_{ar}$), 129.9 (2 x *C*$_{ar}$), 122.9 (2 x *C*$_{ar}$), 122.6 (2 x *C*$_{ar}$), 41.9 (*C*H$_2$NH). **HRMS-ESI$^-$ (m/z):** [M+H]$^+$ cald. for: $C_{14}H_{13}N_3O_2H$, 256.1081; found: 256.1077.

Fluorescence Polarization-based Assays

All FP-based assays were performed in black 96-well microtiter plates (Greiner) and FP was measured as milipolarization (mP) units on a plate reader (Tecan Spark 20M); settings: excitation: 485 nm; emission: 530 nm; gain: optimal; Z-position: calculated from control well (0% inhibition: tracer and protein in assay buffer); number of flashes: 30; G-factor: 1.000. The data evaluation was done, using GraphPad Prism 6 software.

The concentration of the peptides was calculated through UV/Vis measurements (Beckman-Coulter DU800, AMPB-containing peptides (for *trans* isomer) $\mathcal{E}_{335}$ = 25000 L mol^{-1} cm^{-1},[6] fluorescently tagged peptide **22** $\mathcal{E}_{494}$ = 76900 L mol^{-1} cm^{-1}).[11]

Saturation Binding Experiments

Dilutions of the WDR5Δ23 protein (2.0-0 µM, 25µL) were added to 100 µL of a fixed concentration (20 nM) of the fluorescently tagged tracer peptide **22**. Both, protein and tracer, were dissolved in the assay buffer (0.1 M phosphate buffer pH = 6.5, 25 mM KCl, 0.01% Triton) with 4% of DMSO. The total volume was 125 µL. Each assay had two controls: blank (without tracer and protein) and tracer only. The plates were incubated at rt on a shaker (Edmund Bühler TiMix Control TH15) to reach equilibrium and the mP values were measured after 3 h. The measurements were done in triplicate and the standard deviation was calculated. The K_d-value was calculated by converting the mP values into their corresponding anisotropy (A) values. These values were normalized and plotted versus the respective concentrations of the protein with a nonlinear regression according to the following equation:[12] $A = A_f + (A_b - A_f)\,\frac{(L_T + K_d + R_T) - \sqrt{[(L_T - K_d - R_T)^2 - 4 L_T R_T]}}{2\,L_T}$

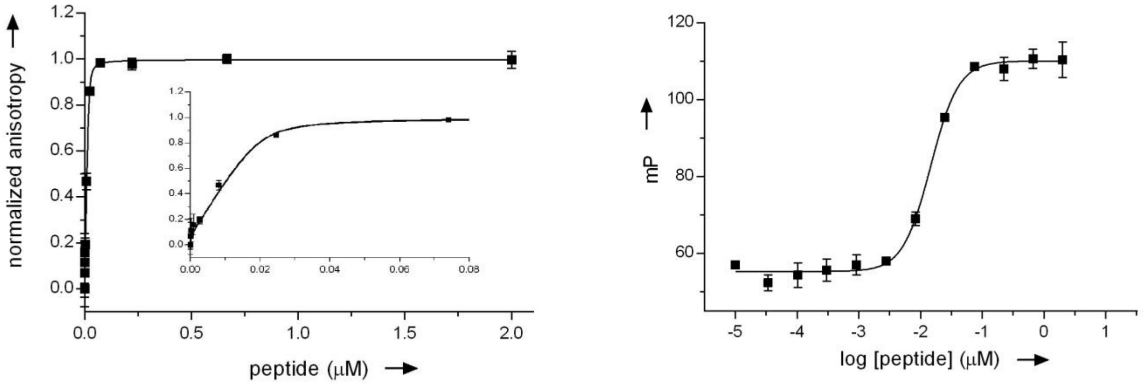

Figure S26. Left: saturation binding curve of peptide **18** obtained from the normalized anisotropy (20 nM tracer). The small picture is zoomed in to show the small x-values. Right: dose-response curve for the same experiment and conditions.

The dose-response curve provides the EC_{50} value of the tracer peptide **22**. To obtain the K_d value, the mP values had to be converted into their corresponding anisotropy (A) values, which were plotted versus the respective concentrations of the protein with a nonlinear regression according to the equation described above. The obtained values are the following: EC_{50} = 0.0145 ± 0.002 µM; K_d = 0.00104 ± 0.0005 µM.

Corroboration of Absence of Quenching Artefacts

To ultimately ensure, that the AMPB molecule incorporated into the peptide backbone does not have any quenching effects in the used FP-based assay, some controls were performed.

Thus, the anisotropy of a 20 nM solution of fluorescein dissolved in 0.1 M phosphate buffer pH = 6.5 with increasing concentrations of AMPB **26** dissolved in DMSO, was measured in triplicate. This measurement was repeated three times, independently. Figure S27 exemplifies one measurement. As shown in such figure, the increase of the amount of AMPB has no influence on the measured milli Polarization values.

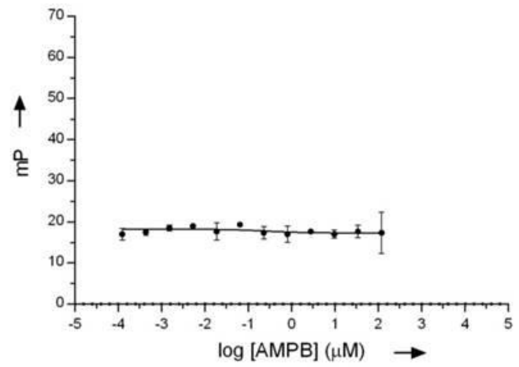

Figure S27. Measured mP values of a 20 nM fluorescein solution with increasing AMPB concentrations.

Furthermore, we also performed a control experiment where 120 µL of a pre-incubated complex solution of WDR5Δ23 and the tracer peptide **22** in assay buffer (0.1 M phosphate buffer pH = 6.5, 25 mM KCl, 0.01% Triton) plus peptide **3** were added to 5 µL of increasing dilutions of AMPB **26** in DMSO, reaching the final concentrations of 72 nM for WDR5Δ23, 20 nM for the tracer peptide and 0.5 µM for the peptide **3**. Again, the experiment was done in triplicate and performed three times independently. As illustrated in Figure S28, AMPB did not affect the milli Polarization values, which are constant during the whole experiment and the same as the previous value without extra AMPB addition as it is shown the overlay with the competitive Fluorescence Polarization-based Binding Assay (Figure S28 right).

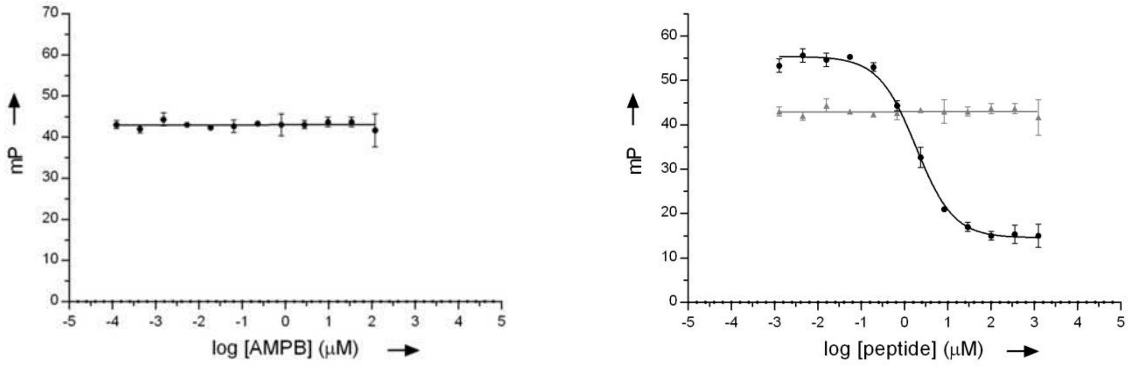

Figure S28. Left: measured mP values of FP-assay (20 nM tracer, 72 nM WDR5, 0.5 µM peptide **3**) with increasing concentrations of AMPB. Right: Overlay of dose response curve of peptide **3**, with left picture.

Competitive Fluorescence Polarization-based Binding Assays

The binding affinities of both isomers of each peptide were measured separately. The peptides were dissolved in DMSO. In order to calculate the binding affinity of the *trans* isomers, the peptide solutions were used directly, without previous irradiation since it was demonstrated that the maximum *trans* ratio is obtained directly from the purification. Contrarily, to determine the binding affinity of the *cis* isomers, the peptide solutions were irradiated at 366 nm for 2 h in a transparent glass vial, previously to the assay. Afterwards, the whole assay had to be done in darkness to prevent $cis \rightarrow trans$ relaxation. 120 µL of a preincubated complex solution of WDR5**Δ**23 and the tracer peptide **22** in assay buffer (0.1 M phosphate buffer pH = 6.5, 25 mM KCl, 0.01% Triton) were added to 5 µL of dilutions of the respective AMPB-containing peptide in DMSO, reaching the final concentrations of 72 nM for WDR5**Δ**23 and 20 nM for the tracer peptide **22**. Three control wells were included in each plate: blank (without tracer and protein), 100% inhibition (tracer only) and 0% inhibition (protein and tracer). The plates were incubated on a shaker (Edmund Bühler TiMix Control TH15) at rt for 5 h and the mP values were recorded. K_i-values were calculated using the following equation described previously by WANG et al. and the corresponding webpage provided by them.[13] $K_i = \frac{[I]_{50}}{\frac{[L]_{50}}{K_d} + \frac{[P]_0}{K_d} + 1}$

To verify that the used assay conditions do not influence the *cis*/*trans* ratio, a control well (in triplicate) with 5 µL of the respective peptide in DMSO irradiated at 366 nm (*cis*), as well as the non-irradiated peptide (*trans*), each diluted with 120 µL assay buffer were included in each plate. Before and after the assay, HPLC chromatograms of such controls were recorded. We did not observe any influence on *cis*/*trans* ratio due to the assay conditions. Table S1 summarizes the *cis*/*trans* ratio of all AMPB-containing peptides after irradiation at 366 nm (*cis*) for 1 h of the respective peptide solution in DMSO and the non-irradiated peptide (*trans*) before the use in the FP-based assays, as well as the *cis*/*trans* ratios of the control wells after the FP-based assay.

Furthermore, to rule out any photo-degradative effects, we not just measured the inhibition constants of the non-irradiated control peptide **3**, but also of the irradiated peptide **3** at both, 366 nm and 430 nm. HPLC-chromatograms after the irradiation but before the FP-based assay were recorded as well as after the FP-based. These, and the chromatograms of the AMPB-containing peptides, are shown in Figure S29 to Figure S58.

Table S1. *cis*/*trans* ratio of the AMPB-containing peptides after purification, after irradiation at 366 nm and after the FP-assay.

peptide sequence	*cis*/*trans* ratio of non-irradiated peptides before FP-assay	*cis*/*trans* ratio of irradiated peptides (366 nm for 1 h) before FP-assay	*cis*/*trans* ratio of non-irradiated peptides after FP-assay	*cis*/*trans* ratio of irradiated peptides (366 nm) after FP-assay
H_2N-S**X**ARAEVHLRKS **4**	12:88	75:25	20:80	71:29
H_2N-S**X**RAEVHLRKS **5**	8:92	75:25	9:91	79:21
H_2N-SAR**X**EVHLRKS **6**	8:92	79:21	11:89	81:19
H_2N-SARA**X**VHLRKS **7**	9:91	85:15	10:90	80:20
H_2N-SARAE**X**HLRKS **8**	10:90	80:20	11:89	79:21
H_2N-SARAEV**X**LRKS **9**	9:91	79:21	9:91	79:21
H_2N-SARAEVH**X**RKS **10**	10:90	81:19	11:89	81:19
H_2N-SARAEVHL**X**KS **11**	7:93	83:17	7:93	83:17
H_2N-SARAEVHLR**X**S **12**	15:85	79:21	15:85	80:20
H_2N-SARA**X**HLRKS **13**	10:90	79:21	12:88	89:21
Ac-ARA**X**VHLRKS **14**	13:87	87:13	13:87	80:20
Ac-ARA**X**VY **15**	10:90	79:21	10:90	79:21
H_2N-SARA**X**VHLRKS$(R)_8$ **16**	12:88	84:16	10:90	80:20
H_2N-SARA**X**VHLRKS $(RAhxR)_4$AhxB **17**	4:96	82:18	4:96	78:22

X = AMPB, B = *β*-alanine

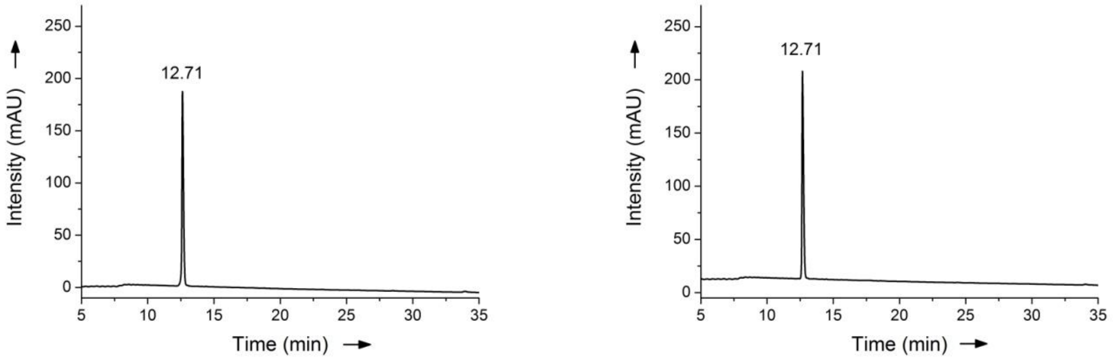

Figure S29. HPLC chromatograms of peptide **3** (H_2N-SARAEVHLRKS-NH_2) before the FP-based assay. Left: irradiated at 366 nm. Right: irradiated at 430 nm.

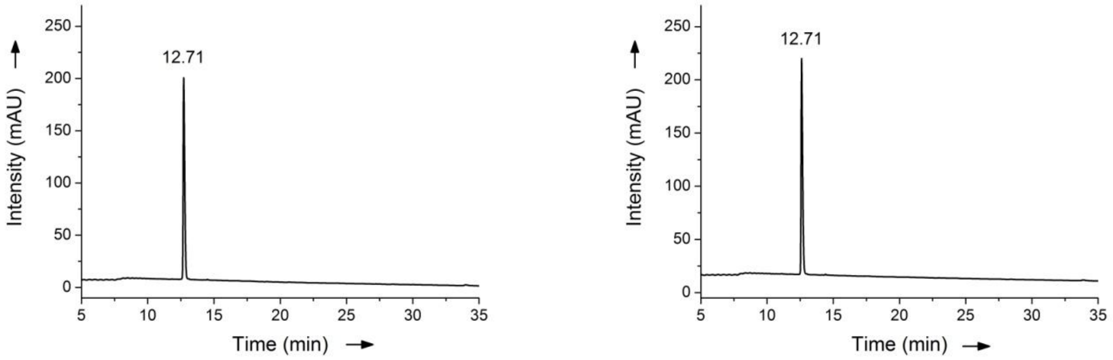

Figure S30. HPLC chromatograms of peptide **3** (H_2N-SARAEVHLRKS-NH_2) after the FP-based assay. Left: irradiated at 366 nm. Right: irradiated at 430 nm.

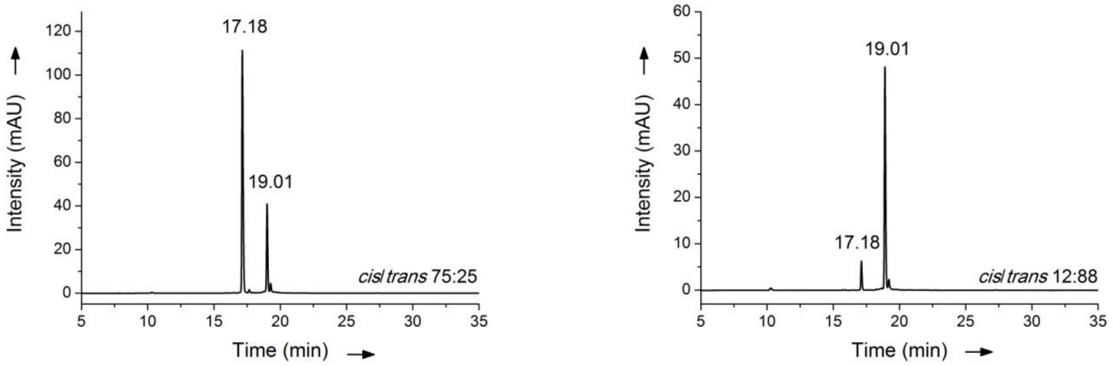

Figure S31. HPLC chromatograms of peptide **4** (H_2N-S-AMPB-ARAEVHLRKS-NH_2) before the FP-based assay. Left: irradiated peptide at 366 nm. Right: non-irradiated peptide.

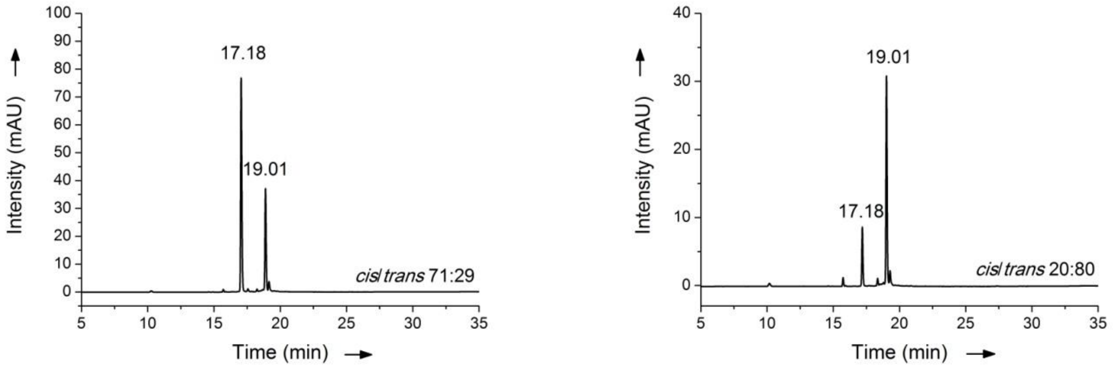

Figure S32. HPLC chromatograms of peptide **4** (H_2N-S-AMPB-ARAEVHLRKS-NH_2) after the FP-based assay. Left: irradiated peptide at 366 nm Right: non-irradiated peptide.

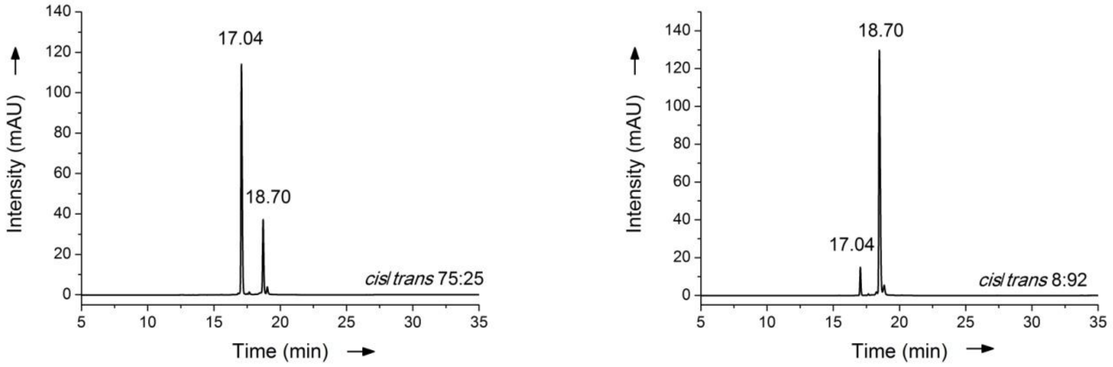

Figure S33. HPLC chromatograms of peptide **5** (H_2N-S-AMPB-RAEVHLRKS-NH_2) before the FP-based assay. Left: irradiated peptide at 366 nm. Right: non-irradiated peptide.

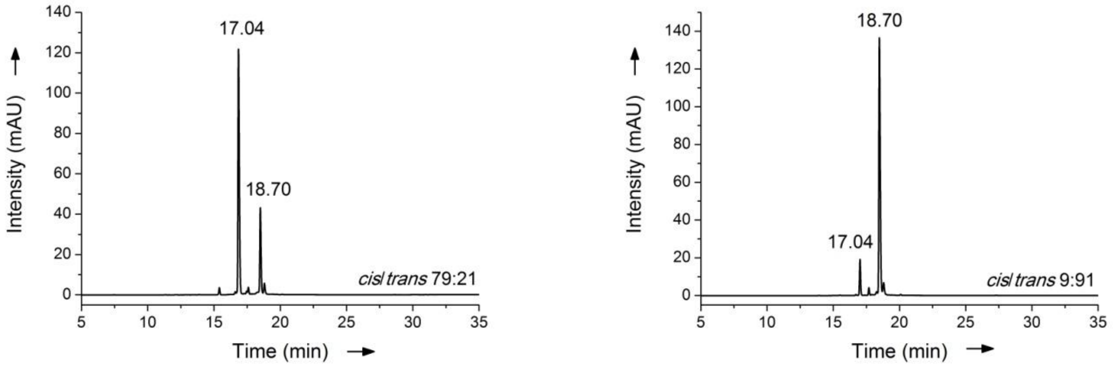

Figure S34. HPLC chromatograms of peptide **5** (H_2N-S-AMPB-RAEVHLRKS-NH_2) after the FP-based assay, left: irradiated peptide at 3666 nm (*cis*). Right: non-irradiated peptide (*trans*).

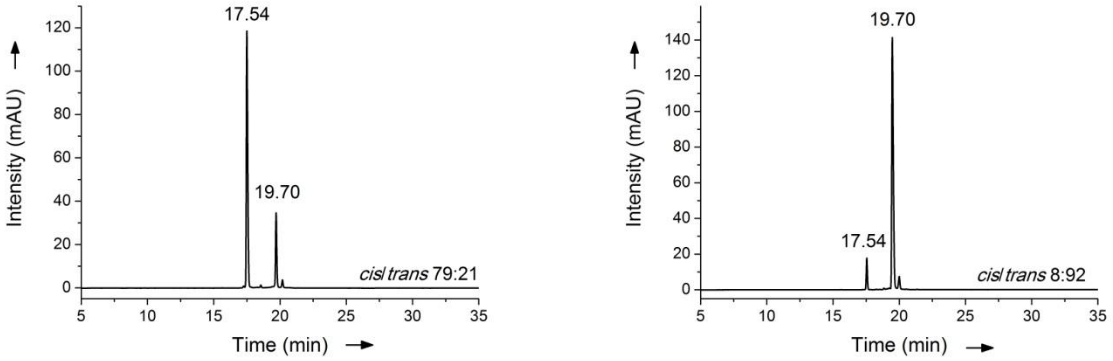

Figure S35. HPLC chromatograms of peptide **6** (H_2N-SAR-AMPB-EVHLRKS-NH_2) before the FP-based assay. Left: irradiated peptide at 366 nm. Right: non-irradiated peptide.

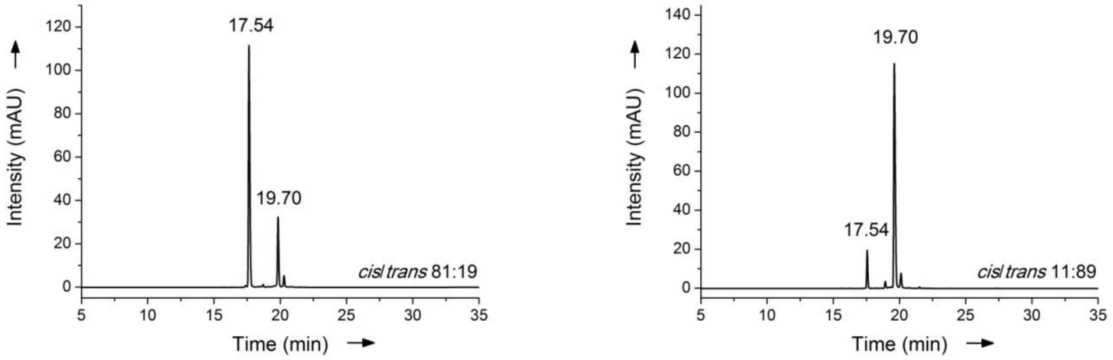

Figure S36. HPLC chromatograms of peptide **6** (H_2N-SAR-AMPB-EVHLRKS -NH_2) after the FP-based assay. Left: irradiated peptide at 366 nm. Right: non-irradiated peptide.

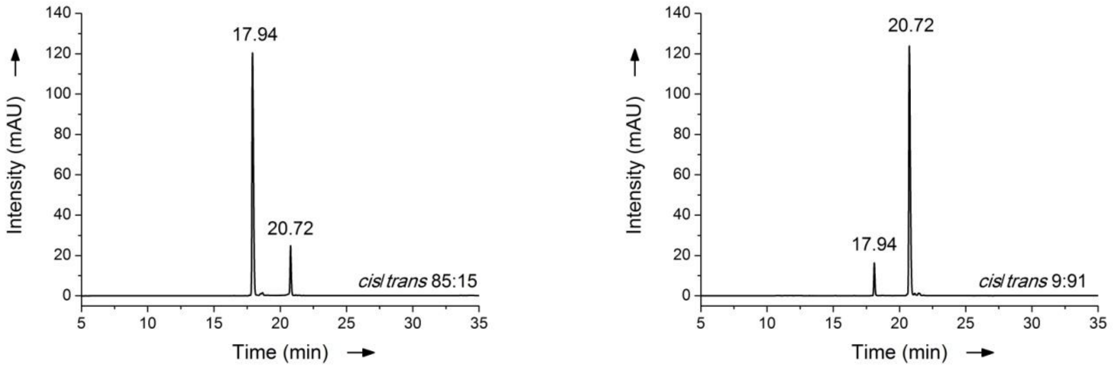

Figure S37. HPLC chromatograms of peptide **7** (H_2N-SARA-AMPB-VHLRKS-NH_2) before the FP-based assay. Left: irradiated peptide at 366 nm. Right: non-irradiated peptide.

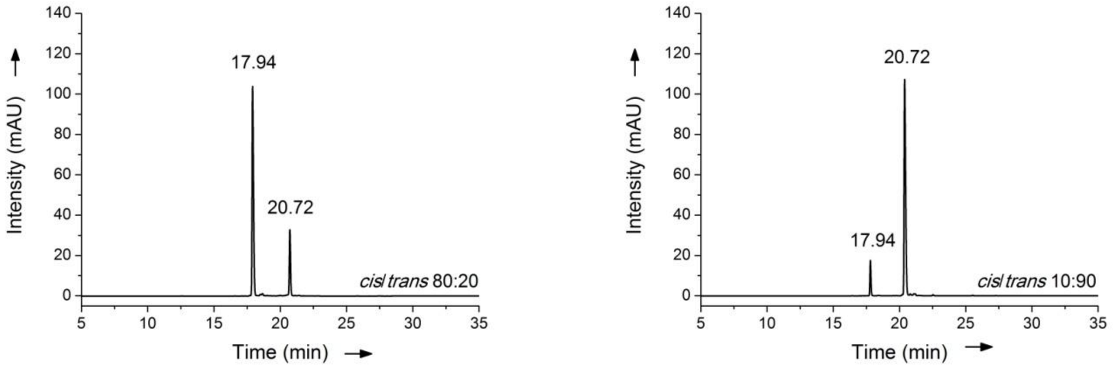

Figure S38. HPLC chromatograms of peptide **7** (H_2N-SARA-AMPB-VHLRKS-NH_2) after the FP-based assay. Left: irradiated peptide at 366 nm. Right: non-irradiated peptide.

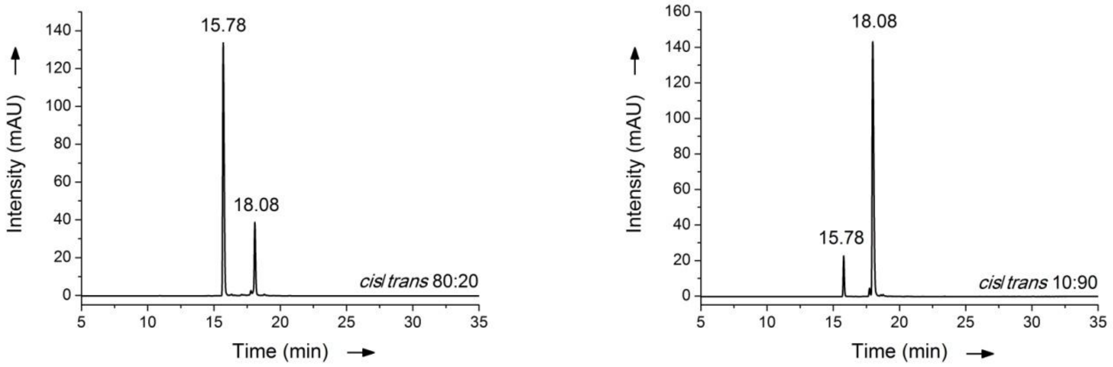

Figure S39. HPLC chromatograms of peptide **8** (H_2N-SARAE-AMPB-HLRKS-NH_2) before the FP-based assay. Left: irradiated peptide at 366 nm. Right: non-irradiated peptide.

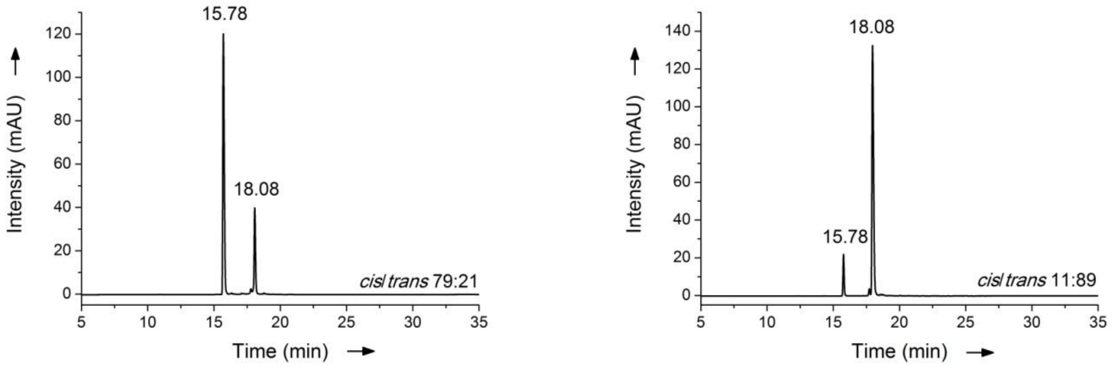

Figure S40. HPLC chromatograms of peptide **8** (H_2N-SARAE-AMPB-HLRKS-NH_2) after the FP-based assay. Left: irradiated peptide at 366 nm. Right: non-irradiated peptide.

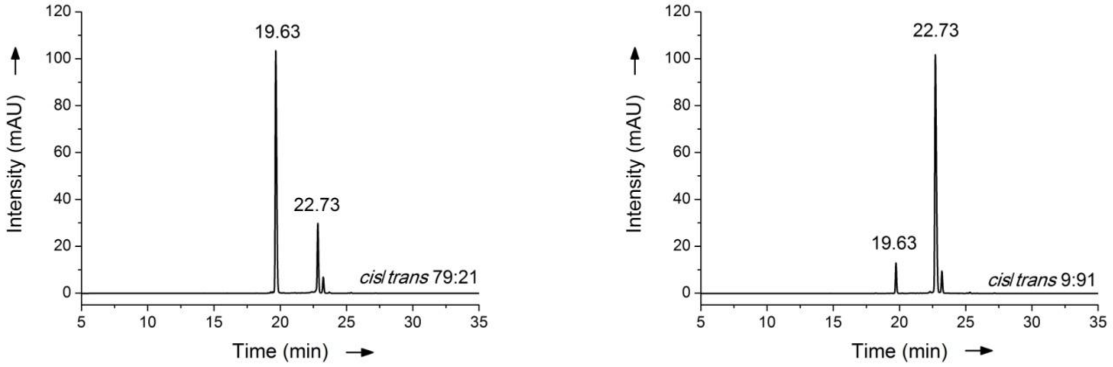

Figure S41. HPLC chromatograms of peptide **9** (H_2N-SARAEV-AMPB-LRKS-NH_2) before the FP-based assay. Left: irradiated peptide at 366 nm. Right: non-irradiated peptide.

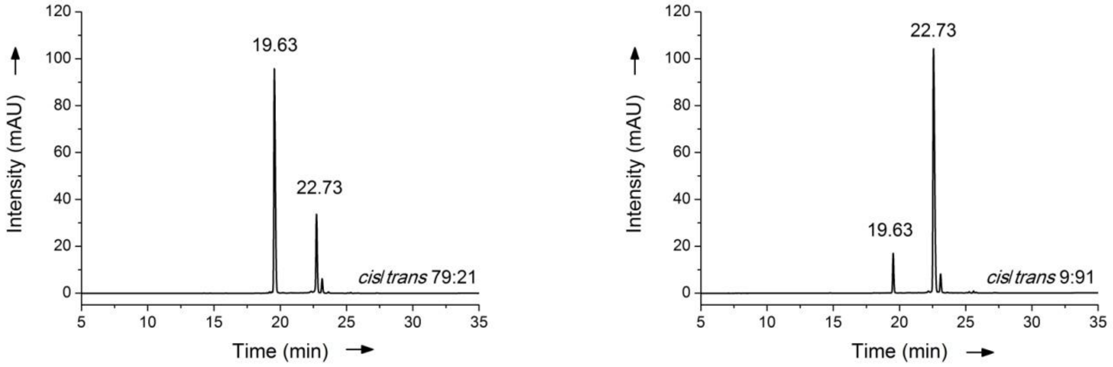

Figure S42. HPLC chromatograms of peptide **9** (H_2N-SARAEV-AMPB-LRKSNH$_2$) after the FP-based assay. Left: irradiated peptide at 366 nm. Right: non-irradiated peptide.

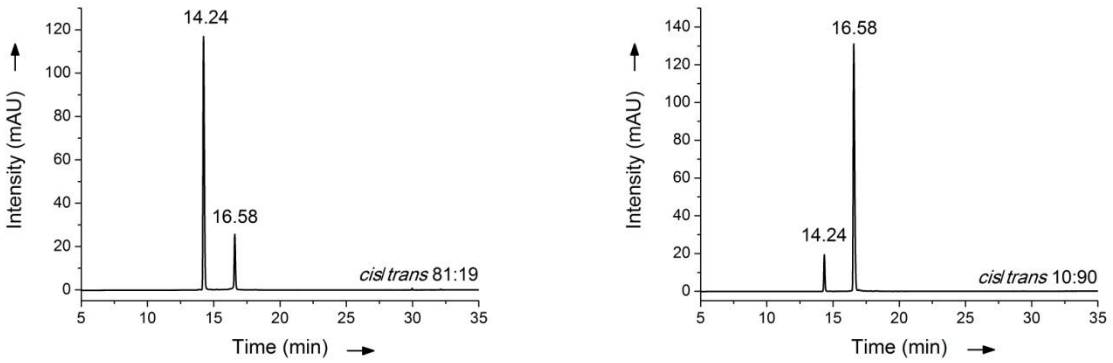

Figure S43. HPLC chromatograms of peptide **10** (H_2N-SARAEVH-AMPB-RKS-NH_2) before the FP-based assay. Left: irradiated peptide at 366 nm. Right: non-irradiated peptide.

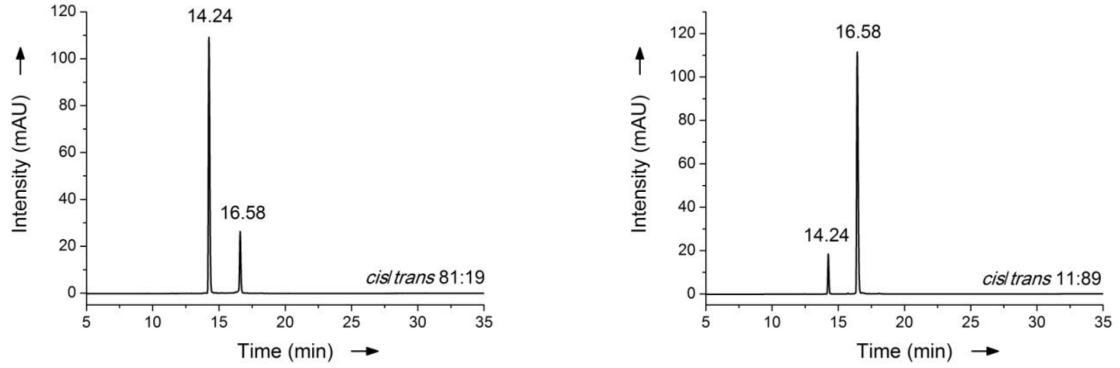

Figure S44. HPLC chromatograms of peptide **10** (H_2N-SARAEVH-AMPB-RKS-NH_2) after the FP-based assay. Left: irradiated peptide at 366 nm. Right: non-irradiated peptide.

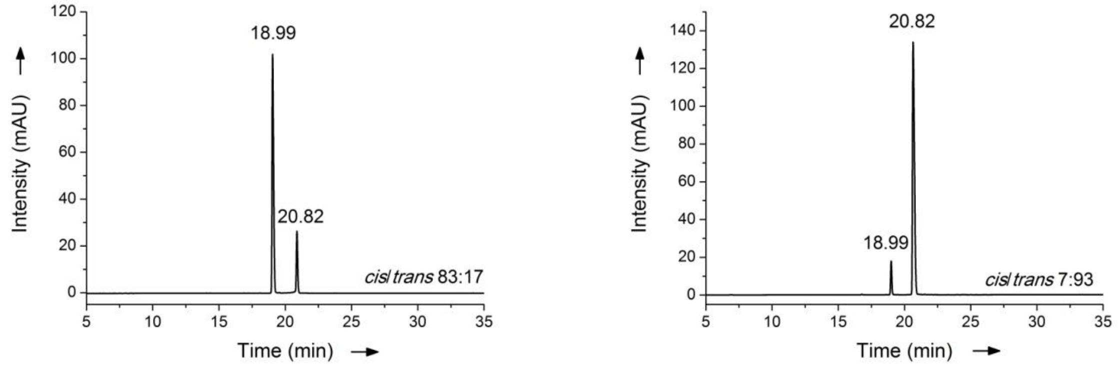

Figure S45. HPLC chromatograms of peptide **11** (H_2N-SARAEVHL-AMPB-KS-NH_2) before the FP-based assay. Left: irradiated peptide at 366 nm. Right: non-irradiated peptide.

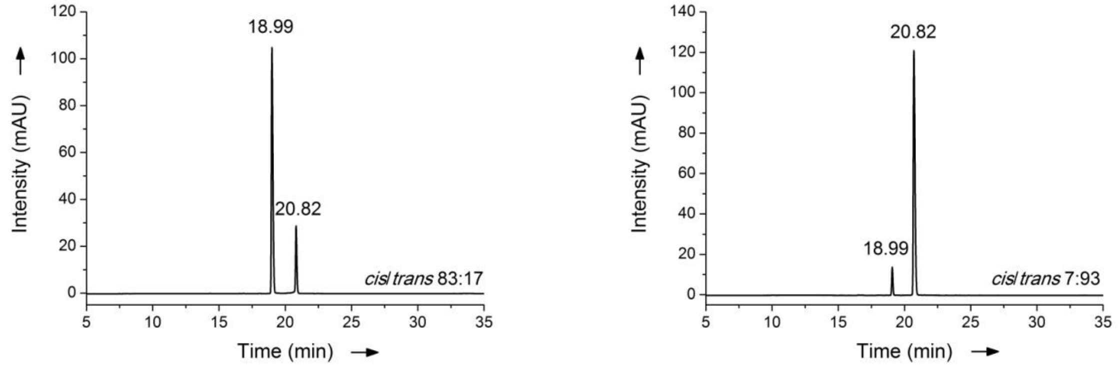

Figure S46. HPLC chromatograms of peptide **11** (H_2N-SARAEVHL-AMPB-KS-NH_2) after the FP-based assay. Left: irradiated peptide at 366 nm. Right: non-irradiated peptide.

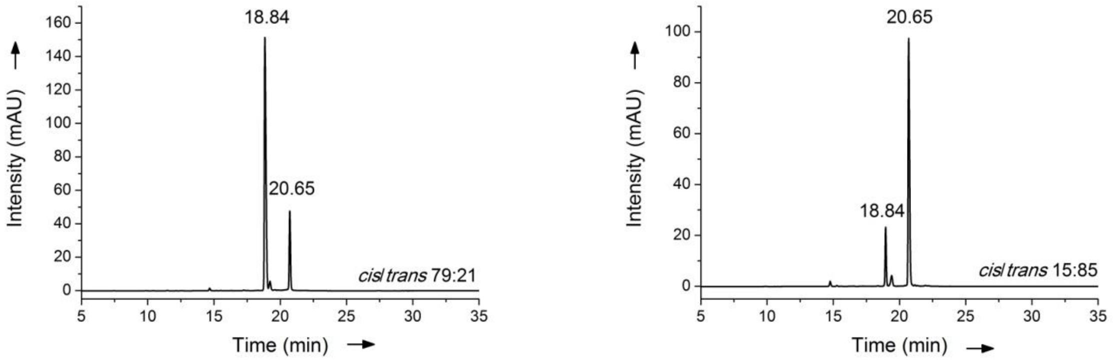

Figure S47. HPLC chromatograms of peptide **12** (H_2N-SARAEVHLR-AMPB-S-NH_2) before the FP-based assay. Left: irradiated peptide at 366 nm. Right: non-irradiated peptide.

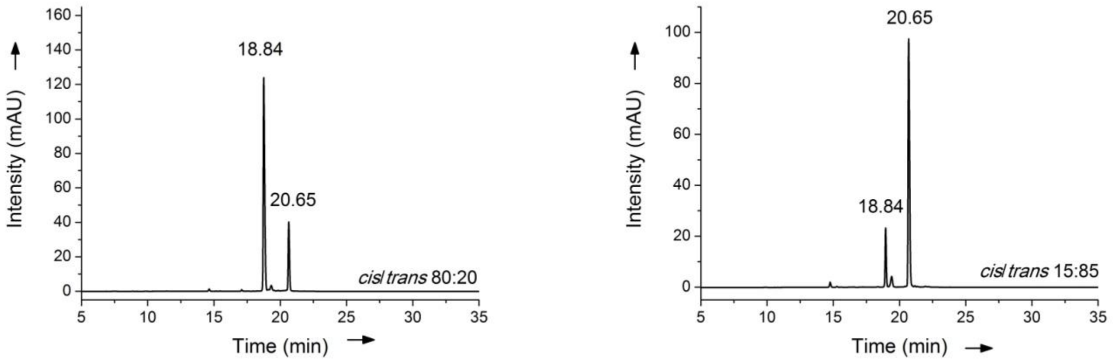

Figure S48. HPLC chromatograms of peptide **12** (H_2N-SARAEVHLR-AMPB-S-NH_2) after the FP-based assay. Left: irradiated peptide at 366 nm. Right: non-irradiated peptide.

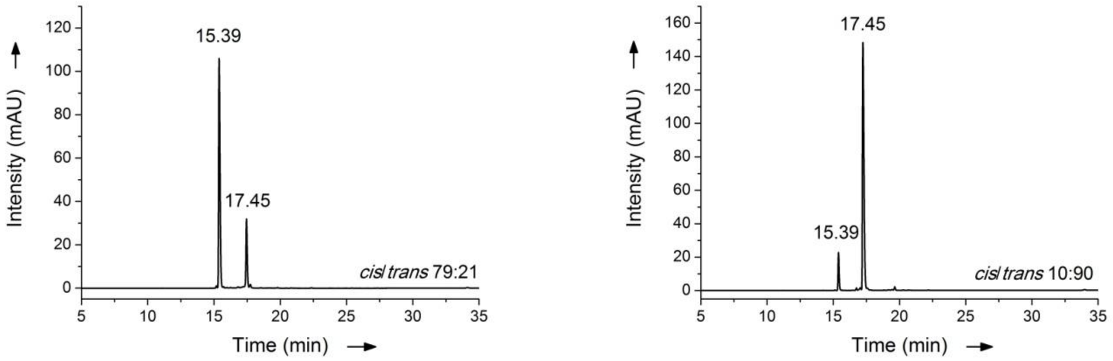

Figure S49. HPLC chromatograms of peptide **13** (H_2N-SARA-AMPB-HLRKS-NH_2) before the FP-based assay. Left: irradiated peptide at 366 nm. Right: non-irradiated peptide.

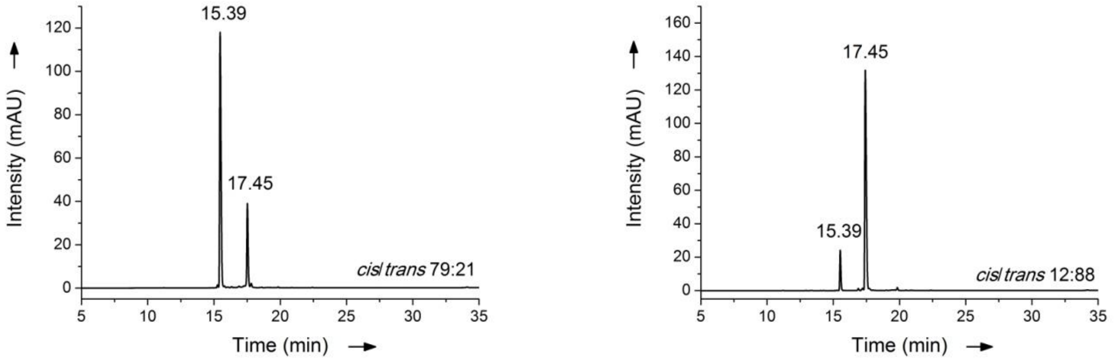

Figure S50. HPLC chromatograms of peptide **13** (H_2N-SARA-AMPB-HLRKS-NH_2) after the FP-based assay. Left: irradiated peptide at 366 nm. Right: non-irradiated peptide.

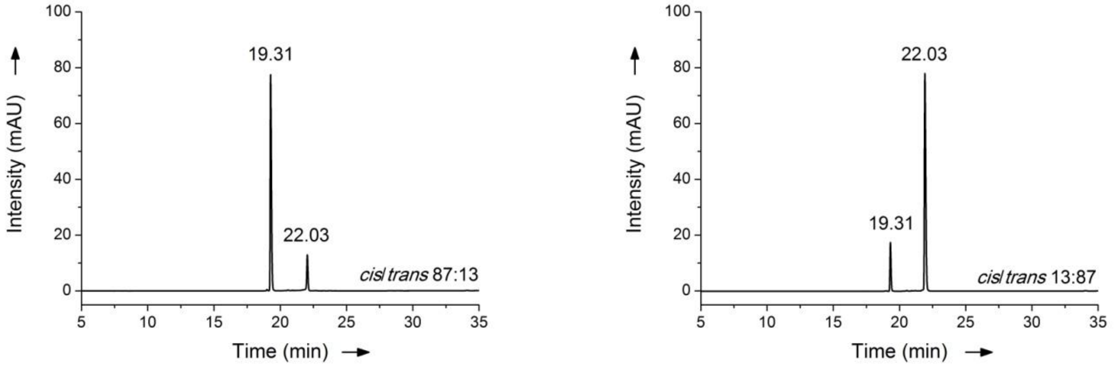

Figure S51. HPLC chromatograms of peptide **14** (Ac-ARA-AMPB-VHLRKS-NH_2) before the FP-based assay. Left: irradiated peptide at 366 nm. Right: non-irradiated peptide.

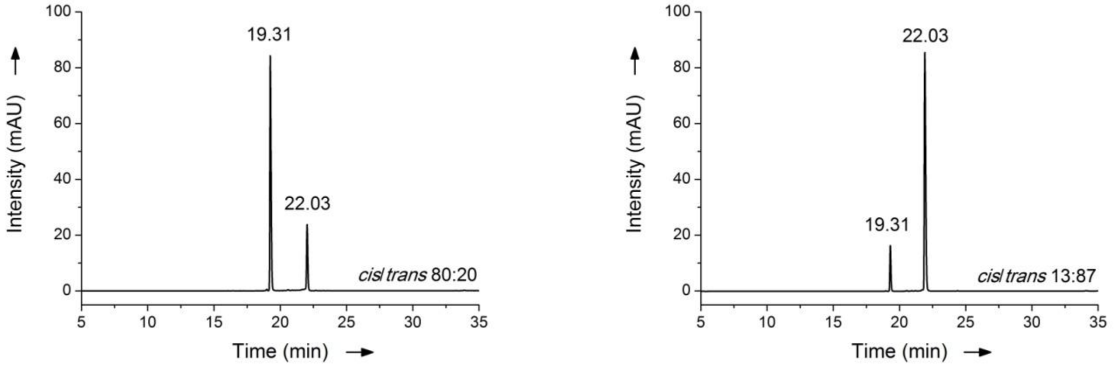

Figure S52. HPLC chromatograms of peptide **14** (Ac-ARA-AMPB-VHLRKS-NH_2) after the FP-based assay. Left: irradiated peptide at 366 nm. Right: non-irradiated peptide.

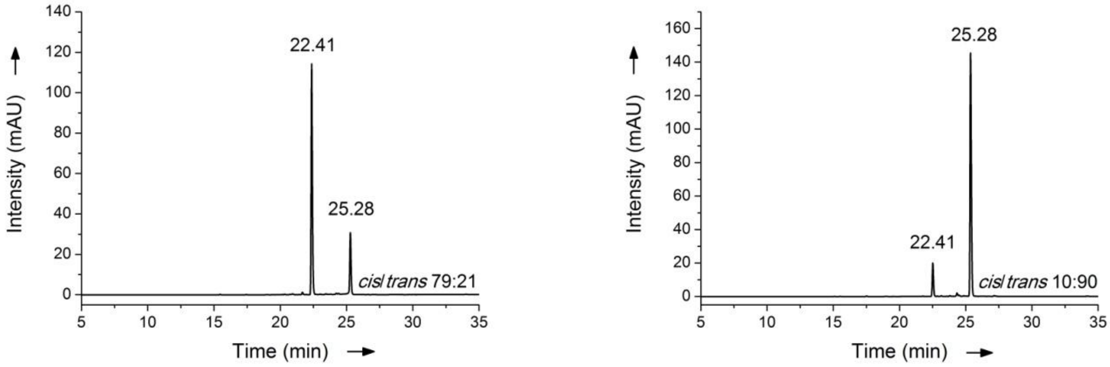

Figure S53. HPLC chromatograms of peptide **15** (Ac-ART-AMPB-VY-NH_2) before the FP-based assay. Left: irradiated peptide at 366 nm. Right: non-irradiated peptide.

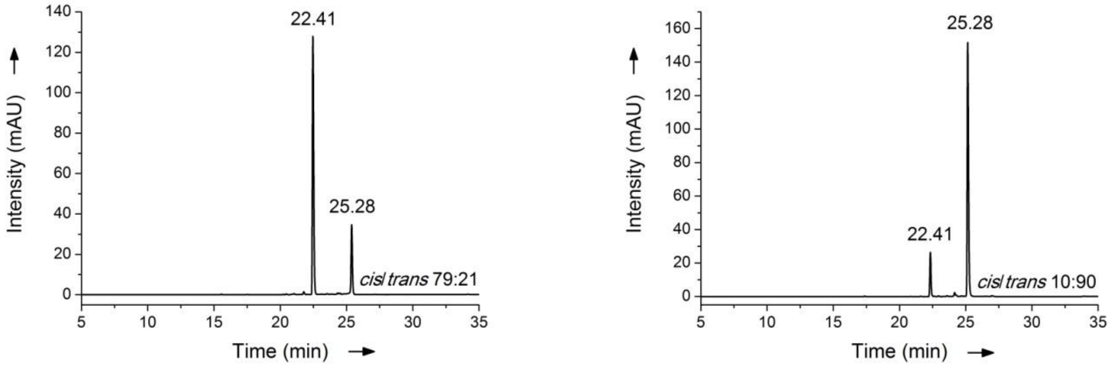

Figure S54. HPLC chromatograms of peptide **15** (Ac-ART-AMPB-VY-NH_2) after the FP-based assay. Left: irradiated peptide at 366 nm. Right: non-irradiated peptide.

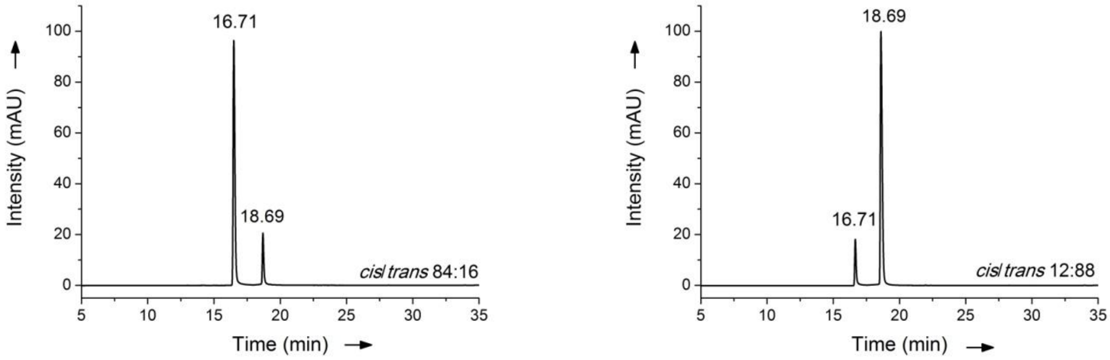

Figure S55. HPLC chromatograms of peptide **16** (H_2N-SARA-AMPB-VHLRKS-R_8-NH_2) before the FP-based assay, left: irradiated peptide at 3666 nm (*cis*). Right: non-irradiated peptide (*trans*).

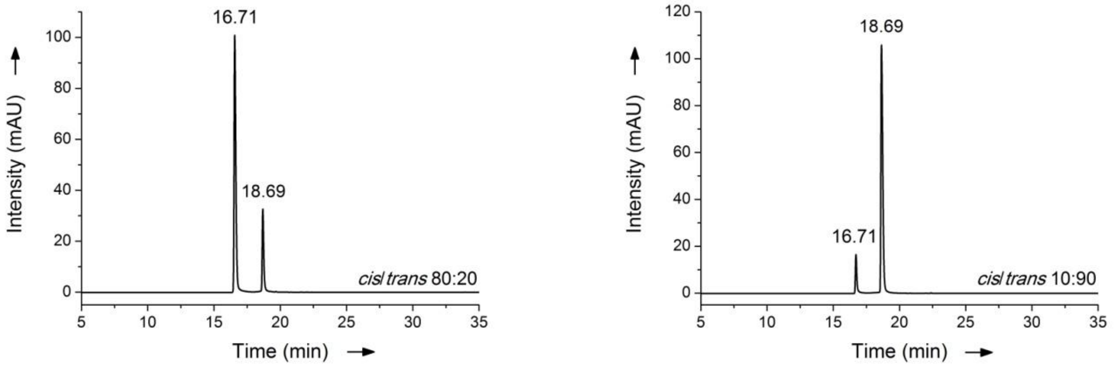

Figure S56. HPLC chromatograms of peptide **16** (H_2N- SARA-AMPB-VHLRKS-R_8-NH2) after the FP-based assay. Left: irradiated peptide at 366 nm. Right: non-irradiated peptide.

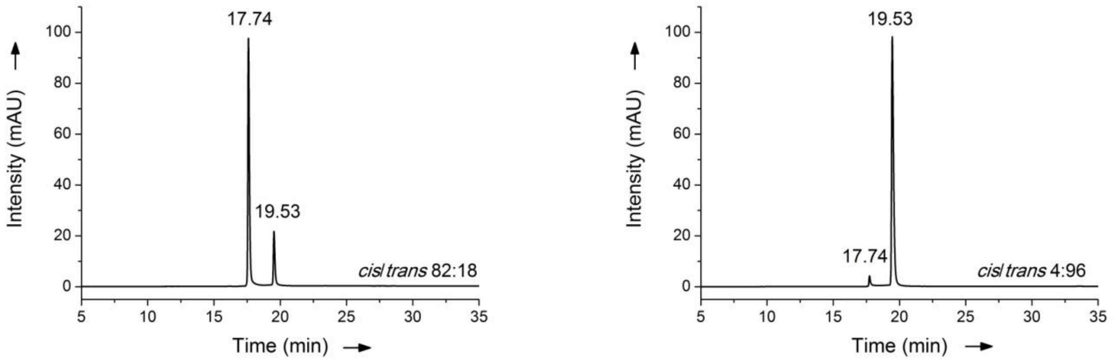

Figure S57. HPLC chromatograms of peptide **17** (H_2N-SARA-AMPB-VHLRKS-(RahxR)$_4$RAhxB-NH_2) before the FP-based assay. Left: irradiated peptide at 366 nm. Right: non-irradiated peptide.

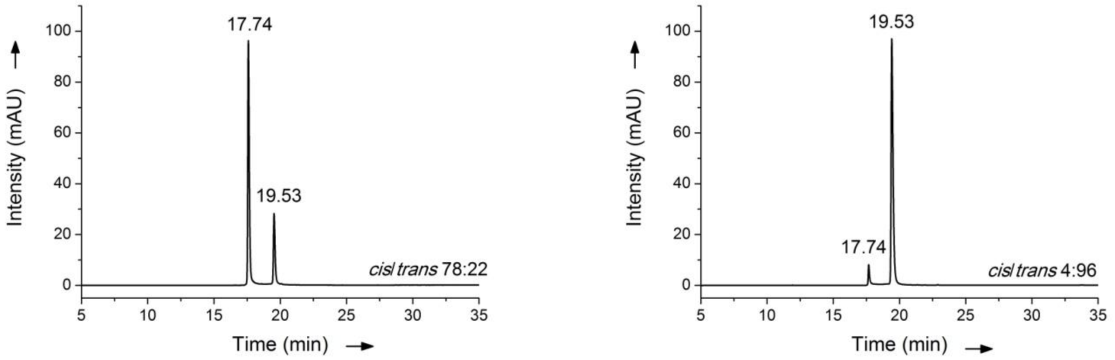

Figure S58. HPLC chromatograms of peptide **17** (H_2N-SARA-AMPB-VHLRKS-(RahxR)$_4$RAhxB-NH_2) after the FP-based assay. Left: irradiated peptide at 366 nm. Right: non-irradiated peptide.

The FP measurements were done in triplicate and the competitive assay of both isomers of every peptide was measured three times independently. Here, the graphs of one measurement are exemplified for each peptide.

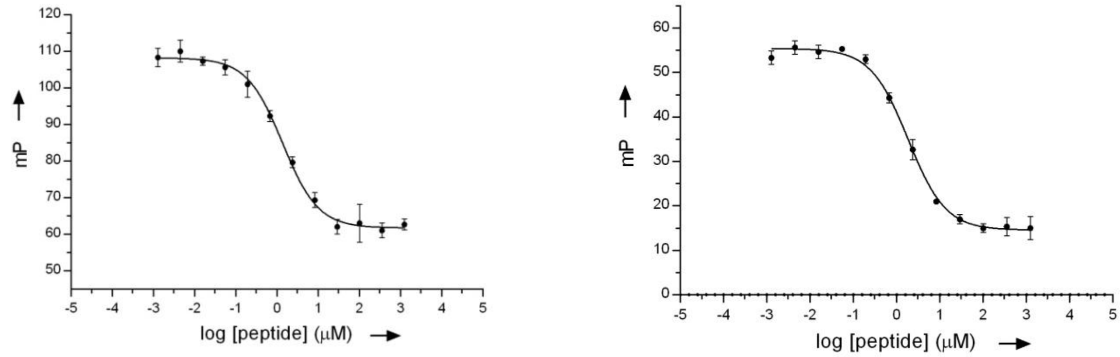

Figure S59. Dose-response curves of: left: peptide **3** (SARAEVHLRKS) non-irradiated, right: peptide **3** (SARAEVHLRKS) irr. at 430 nm.

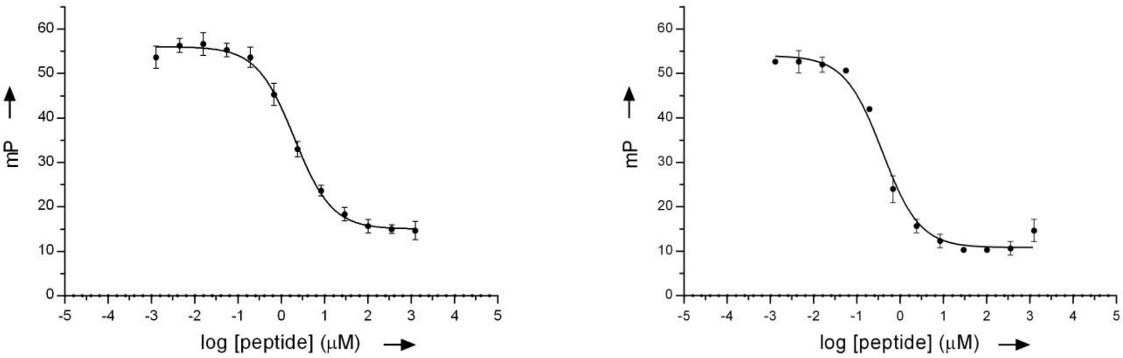

Figure S60. Dose-response curves of: left: peptide **3** (SARAEVHLRKS) irr. at 360 nm, right: peptide **20** (SARAEVHLRKS-R_8).

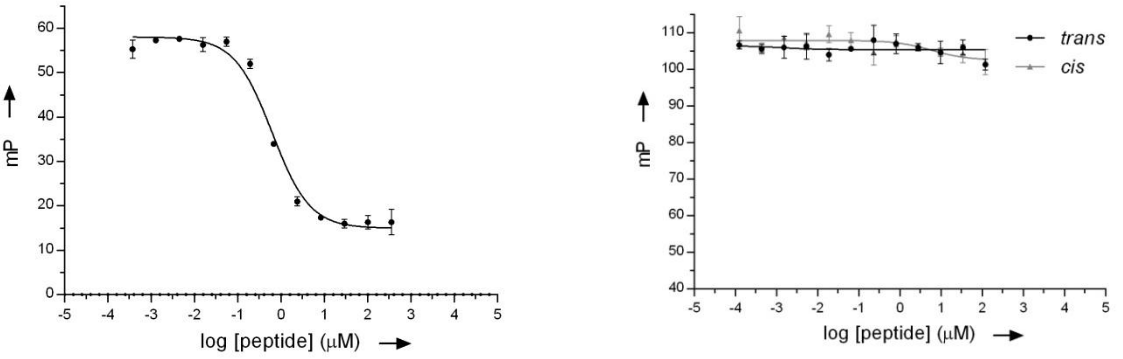

Figure S61. Dose-response curves of: left: **21** (SARAEVHLRKS-$(RAhxR)_4$RAhxB), right: peptide **4** (S-AMPB-ARAEVHLRKS).

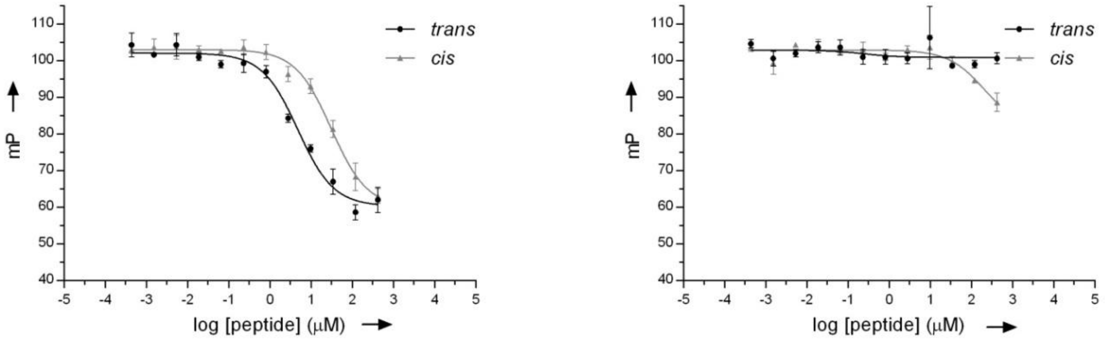

Figure S62. Dose-response curves of: left: peptide **5** (S-AMPB-RAEVHLRKS), right: peptide **6** (SAR-AMPB-EVHLRKS).

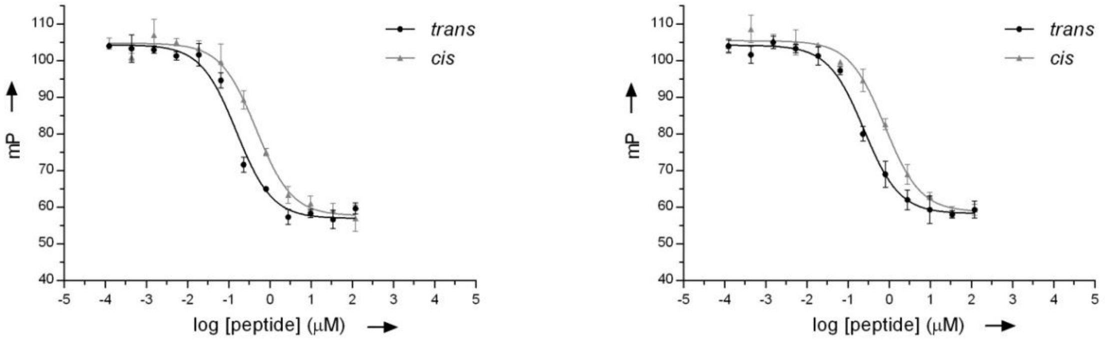

Figure S63. Dose-response curves of: left: peptide **7** (SARA-AMPB-VHLRKS), right: peptide **8** (SARAE-AMPB-HLRKS).

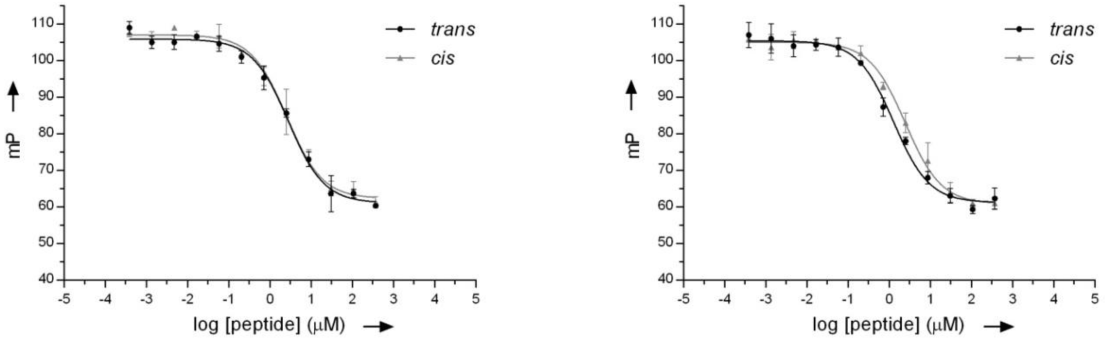

Figure S64. Dose-response curves of: left: peptide **9** (SARAEV-AMPB-LRKS), right: peptide **10** (SARAEVH-AMPB-RKS).

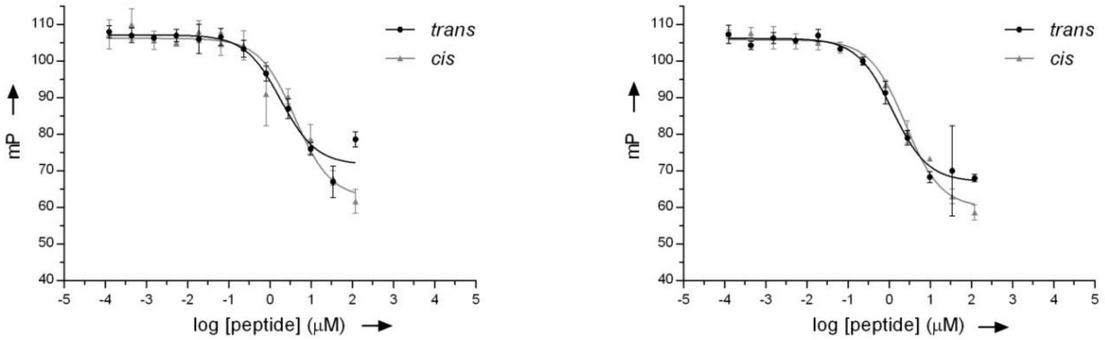

Figure S65. Dose-response curves of: left: peptide **11** (SARAEVHL-AMPB-KS), right: peptide **12** (SARAEVHLR-AMPB-S).

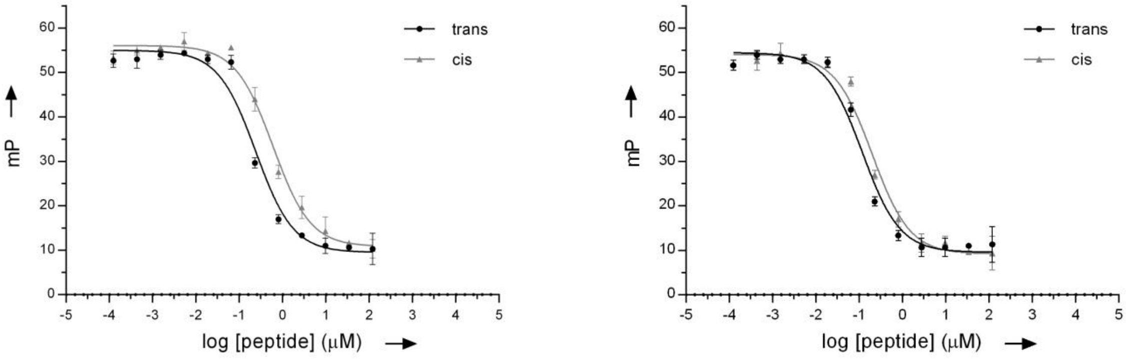

Figure S66. Dose-response curves of: left: peptide **13** (SARA-AMPB-HLRKS), right: peptide **15** (Ac-ART-AMPB-VY).

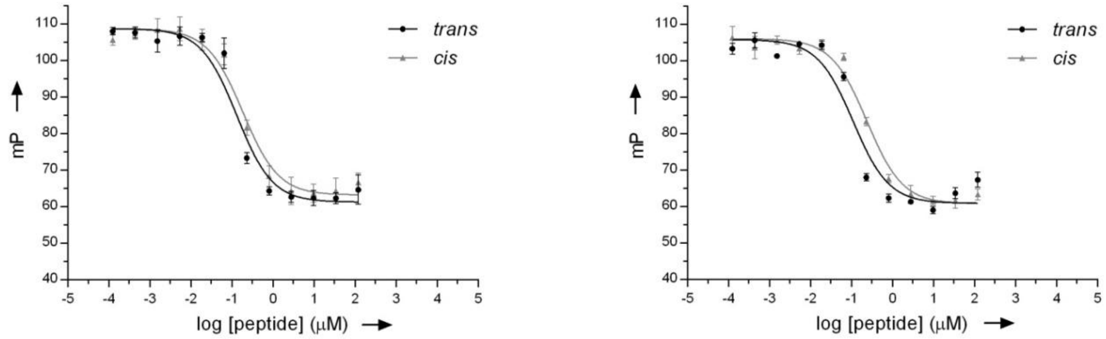

Figure S67. Dose-response curves of: left: peptide **16** (SARA-AMPB-VHLRKS(R)$_8$), right: peptide **17** (SARA-AMPB-VHLRKS(RAhxR)$_4$AhxB).

The mean and the standard deviation of the three independent measurements were calculated and are listed in the Table S2 to Table S4.

Table S2. Mean ± SD of the IC_{50} and K_i values of peptides **3, 20** and **21**.

peptide	IC_{50} [µM]	K_i [nM]
H_2N-SARAEVHLRKS **3**	1.34 ± 0.062	20.0 ± 0.98
H_2N-SARAEVHLRKS-R_8 **20**	0.368 ± 0.02	4.83 ± 0.31
H_2N-SARAEVHLRKS-(RAhxB)$_4$RAhxB **21**	0.612 ± 0.02	8.67 ± 0.32

Table S3. Mean ± SD of the IC_{50} and K_i values of the irradiated peptide **3**.

peptide	IC_{50} [µM] (irr. at 430 nm)	IC_{50} [µM] (irr. at 366 nm)	K_i [nM] (irr. at 430 nm)	K_i [nM] (irr. at 366 nm)
H_2N-SARAEVHLRKS **3**	1.43 ± 0.43	1.61 ± 0.033	23.8 ± 6.16	24.5 ± 0.49

Table S4. Mean ± SD of the IC_{50} and K_i values of AMPB-containing peptides **4-17**.

peptide	IC_{50} *trans* [µM]	IC_{50} *cis* [µM]	K_i *trans* [nM]	K_i *cis* [nM]	[*cis/trans*]
H$_2$N-S**X**ARAEVHLRKS **4**	-	-	-	-	-
H$_2$N-S**X**RAEVHLRKS **5**	3.91 ± 0.83	24.9 ± 5.7	66.8 ± 10	433 ± 73	6.48
H$_2$N-SAR**X**EVHLRKS **6**	-	-	-	-	-
H$_2$N-SARA**X**VHLRKS **7**	0.146 ± 0.021	0.475 ± 0.088	1.25 ± 0.36	6.50 ± 1.4	5.20
H$_2$N-SARAE**X**HLRKS **8**	0.210 ± 0.035	0.461 ± 0.14	2.40 ± 0.60	9.10 ± 2.2	3.79
H$_2$N-SARAEV**X**LRKS **9**	2.53 ± 0.39	2.58± 0.023	40.4 ± 3.9	39.7 ± 0.35	0.990
H$_2$N-SARAEVH**X**RKS **10**	1.15 ± 0.12	1.49 ± 1.3	17.0 ± 1.9	33.3 ± 4.2	1.96
H$_2$N-SARAEVHL**X**KS **11**	2.14 ± 0.28	4.54 ± 0.39	32.8 ± 4.5	70.4 ± 6.6	2.15
H$_2$N-SARAEVHLR**X**S **12**	1.32 ± 0.11	2.64 ± 0.16	19.8 ± 1.7	40.6 ± 2.5	2.05
H$_2$N-SARA**X**HLRKS **13**	0.293 ± 0.05	0.664 ± 0.08	3.63 ± 0.80	9.47 ± 1.2	2.61
Ac-ART**X**VY **15**	0.109 ± 0.01	0.185 ± 0.02	0.767 ± 0.15	2.07 ± 0.24	2.70
H$_2$N-SARA**X**VHLRKS-R$_8$ **16**	0.135 ± 0.005	0.192 ± 0.010	1.18 ± 0.095	2.05 ± 0.17	1.74
H$_2$N-SARA**X**VHLRKS-(RAhxR)$_4$AhxB **17**	0.12 ± 0.025	0.227 ± 0.024	0.925 ± 0.39	2.60 ± 0.36	2.81

X = AMPB

For peptide **7** and additionally for peptide **14**, the exact same experiment was repeated just with lower final concentrations of WDR5**Δ**23 and the tracer peptide **22** (protein: 20 nM, tracer: 5 nM). This experiment was done, because the IC_{50} value is depending on the assay conditions, while the K_i value should be in the same range, independently of the assay conditions.[13] The measurement was also done in triplicate and the peptide was measured three times independently. Here, the graphs of one measurement for each peptide are exemplified (Figure S68).

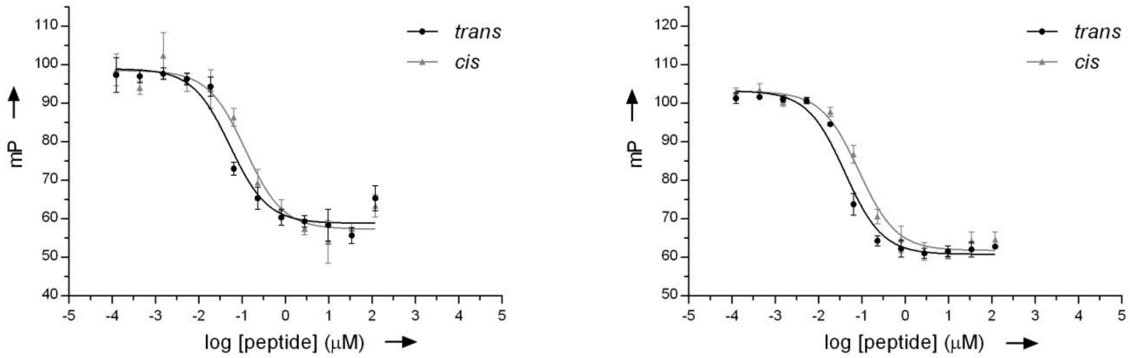

Figure S68. Dose-response curve of, left: peptide **7** (SARA-AMPB-VHLRKS), right: peptide **14** (Ac-ARA-AMPB-VHLRKS) with 5 nM of tracer.

The mean and the standard deviation of these three independent measurements were calculated and are listed in the Table S5.

Table S5. Mean ± SD of the IC_{50} and K_i values of peptides **7** and **14** (with 5 nM tracer).

peptide	IC_{50} *trans* [μM]	IC_{50} *cis* [μM]	K_i *trans* [nM]	K_i *cis* [nM]	[*cis*/*trans*]
H_2N-SARA**X**VHLRKS **7**	0.0570 ± 0.011	0.147 ± 0.014	2.03 ± 0.42	6.37 ± 1.0	3.14
Ac-ARA**X**VHLRKS **14**	0.0450 ± 0.011	0.102 ± 0.015	1.50 ± 0.63	4.50 ± 0.82	3.00

X = AMPB, B = *β*-alanine

Crystal Structure of WDR5Δ23-Peptide 7 Complex

The expressed and purified WDR5Δ23 was mixed in a 4:1 ratio separately with two stock solutions (55.7 mM): *trans/cis* ratio (96:4) and *cis/trans* ratio (81:19) of the peptide **7** (H_2N-SARA-AMPB-VHLRKS-$CONH_2$), what we called ‘state I’ and ‘state II’, giving final concentrations of WDR5 = 229.62 μM (8.5 mg/mL) and peptide **7** = 918.48 μM. Previously, the two isomers of peptide **7** were dissolved in DMSO (55.7 mM) and 2.3 μL of these stock solutions were added to 139.7 μL of the protein in dialysis buffer (25 mM Tris, pH = 8.0, 150 mM NaCl). In the ‘state I’ the peptide solution was used without any irradiation while in the ‘state II’, the solution of peptide **7** was irradiated at 366 nm for 14 h before adding it to the protein and kept in the dark at all times during the crystallization experiments.

Crystallization trials were attempted with available commercial screening solutions with 300nl drop volume of protein-peptide complex at a concentration of 8.5 mg/mL protein and 918.48 μM peptide **7** at 18 °C with a Honeybee robot (Isogen Life Science). The hanging drop

vapor diffusion method was used for crystallization and crystals were obtained with 12.5% w/v PEG 1000, 12.5% w/v PEG 3350, 12.5% v/v MPD, 0.02 M of each carboxylic acid, 0.1 M bicine/Trizma base pH 8.5 (Morpheus screen, Molecular Dimensions). Crystals were flash-frozen directly in the mother liquor solution and no cryoprotectant was necessary as the crystals grew in a cryoprotected condition. All manipulations for the dark state crystals were done under red light.

Diffraction data were collected at the Swiss Light Source (SLS) on the microfocus beamline X06SA. All data were processed using XDS[14] in space group P21212. Data reduction and scaling was done using the CCP4 suite of programs.[15] Data collection and processing statistics are given in Table S6. The structure of the complex of both states of the AMPB-containing peptide **7** in complex with WDR5Δ23 was determined by molecular replacement with PHASER[16] using the coordinates of the previously determined structure of the WDR5Δ23-MLL1 WIN peptide complex (PDB code: 3EG6) as a search model. After an initial rigid body refinement, the structure was further refined with rounds of simulated annealing, energy minimization, and individual *B*-factor refinement with a maximum likelihood target using Phenix.[17] Difference Fourier maps were calculated with Phenix and used to locate electron density corresponding to bound peptide, and the structure was built using COOT.[18] PRODRG[19] was used to generate geometry restraints for the AMPB-containing peptide refinement and Molprobity[20] for structural validation. Final refinement statistics are given in Table S6. All structural figures were generated with Pymol.[21] The coordinates and structure factors (PDB code 5M23 for state I WDR5-AMPB-containing peptide complex and PDB code 5M25 for state II complex) have been deposited to the Protein Data Bank.

Table S6. X-ray data collection and refinement statistics.

	WDR5Δ23-AMPB-containing peptide 7 ('light state')	WDR5Δ23-AMPB-containing peptide 7 ('dark state')
Wavelength (Å)	0.9788	0.9788
Resolution range (Å)	43.05 - 1.97 (2.11-1.97)	42.96 - 2.43 (2.63-2.43)
Space group	P 21 21 2	P 21 21 2
Unit cell (Å, Å, Å ° , °, °)	80.553 86.098 40.407 90.00 90.00 90.00	80.553 86.098 40.407 90.00 90.00 90.00
Total reflections	258124	112937
Unique reflections	20077	17802
Multiplicity	12.7 (12.1)	6.3 (6.3)
Completeness (%)	99.78 (94.4)	98.8 (99.0)
Mean I/sigma(I)	14.4 (5.0)	8.5 (2.2)
Rmerge	0.070 (0.237)	0.090 (0.253)
R-factor	0.1664	0.1795
R-free	0.2143	0.2482
Number of atoms	2471	2469
Protein	2326	2325
ligands	51	44
RMS(bonds)	0.015	0.008
RMS(angles)	1.488	1.196
Ramachandran favored (%)	95.0	94.3
Ramachandran outliers (%)	0.0	0.0
Average B-factor	38.56	15.78

Figure S69 shows an overlay of the whole structures of the superimposed state I and state II of peptide **7**.

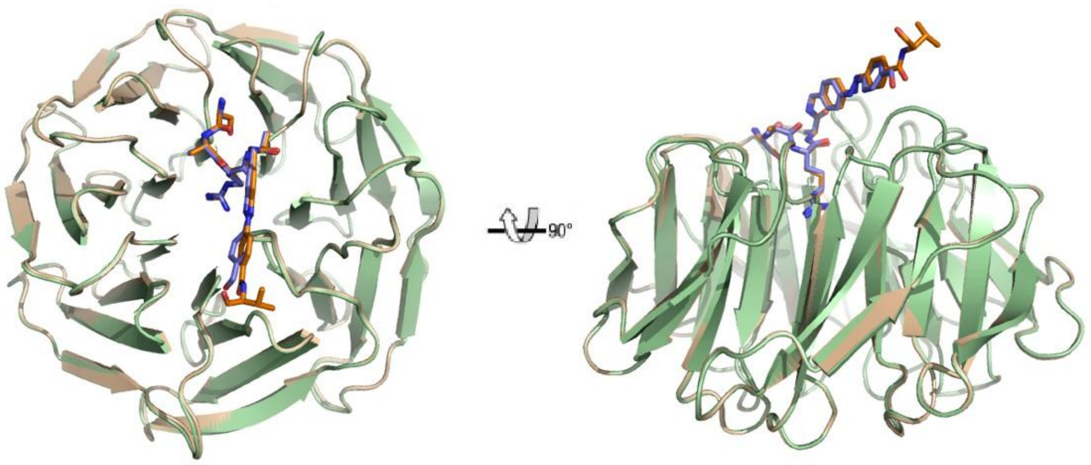

Figure S69. Superposition of the crystal structure of the WDR5Δ23-peptide **7** complex in the state I (peptide: orange, protein: wheat) overlaid with WDR5Δ23-peptide **7** complex in the state II (peptide: blue, protein: green).

The key interactions of peptide **7** with the WDR5 protein in both states agree to the ones seen in the crystal structure of the WIN peptide in complex with WDR5 (PDB code 3EG6).[22,23] Thus, the hydrogen bonds between the Ser and Ala residue of the peptide **7** to Asp-107, as well as the one between the Ser and the Arg main chain residues of the peptide **7** with Ser-91 from WDR5, as well as two indirect hydrogen bonds, mediated by one water molecule, to Val-132 and Asp-107 could be found in our protein-peptide **7** complex. Also, the aromatic side chain of Tyr-260 participates in a water-mediated hydrogen bond to the second Ala residue. Furthermore, the crystal structure confirmed that the Arg side chain of peptide **7** inserts into a central tunnel of the WD40 β-propeller. The guanidinium moiety is sandwiched between the conserved aromatic side chains of Phe-133 and Phe-263, which is stabilized by a cation-π-interaction. Further stabilization is provided by a substantial network of direct and indirect (water mediated) hydrogen bonds between the Arg residue and the amino acids Ser-91, Phe-133, Ser-175, Ser-218 and Cys-261 of WDR5. In the C-terminus, the overlay of the superposition of the the state I AMPB-peptide and the WIN peptide structures shows quite a difference in the location of the two respective peptides. While the His-3769 of the WIN peptide points towards the Phe-149, Asp-172, Pro-173, and Tyr-191 residues of the protein,[22] the azobenzene moiety of our peptide is mostly located outside of the protein structure (Figure 3 in the manuscript). All the interactions between both, the state of peptide **7** with WDR5Δ23 are shown in Figure S70 and Figure S71 and are additionally listed in Table S7 and Table S8.

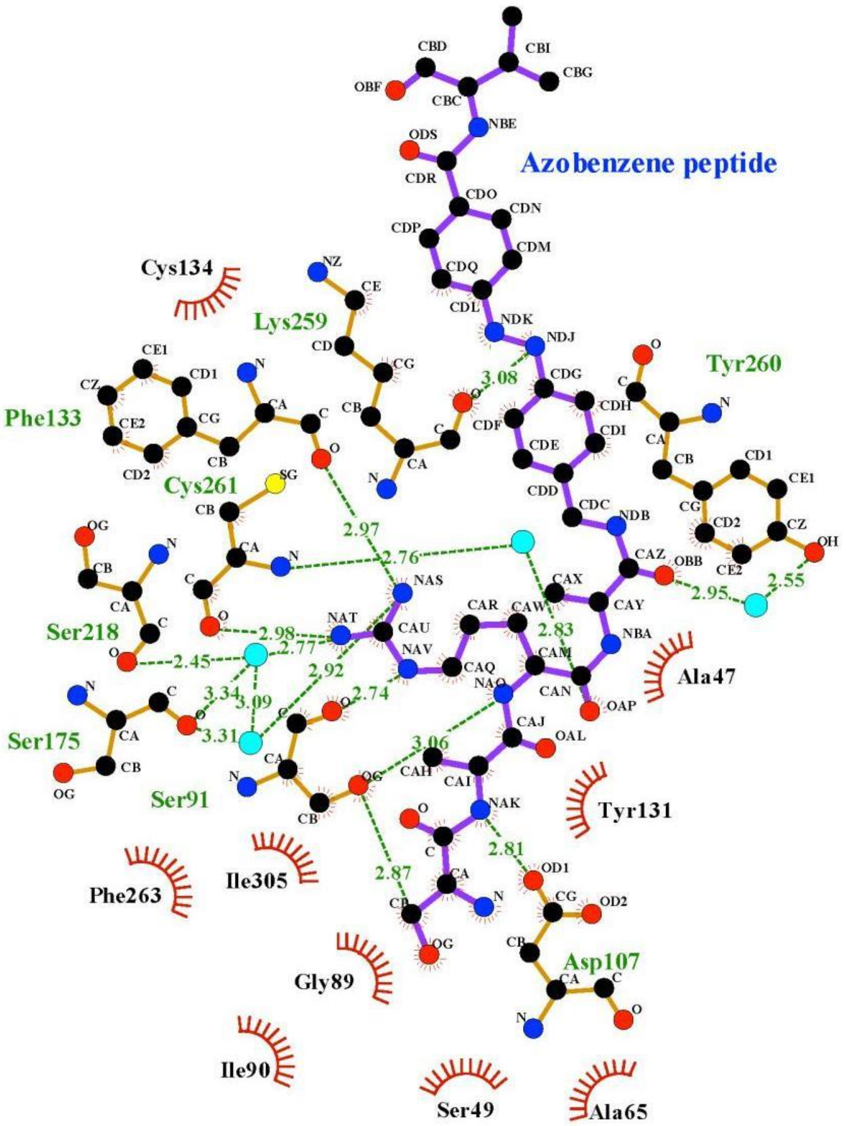

Figure S70. Schematic representation of the key interactions between the AMPB-peptide **7** in the state I and WDR5Δ23. Generated with LIGPLOT.[24]

Table S7. Interactions between the AMPB-peptide **7** in the state I and WDR5Δ23. Interactions marked in red, are strong H-bonds.

peptide		Hydrogen-bond/ Van der Waals partner		
Residue	Atom	WDR5 residue	Atom	Distance (Å)
AMPB5	CDQ	Lys259	O	3.75
AMPB5	CDL	Lys259	CG	3.90
AMPB5	NDK	Lys259	CG	3.69
AMPB5	NDJ	Lys259	O	3.08
			CG	3.17
			CE	3.82
AMPB5	CDG	Lys259	O	3.88
			CG	3.93
			CE	3.83
AMPB5	CDH	Lys259	O	3.81
		Tyr260	CE2	3.82

		Tyr260	CD2	3.43
AMPB5	CDI	Tyr260	CE2	3.34
		Tyr260	CD2	3.59
AMPB5	CDF	Lys259	CE	3.74
AMPB5	CDD	Tyr260	CE2	3.93
Ala4	OBB	Tyr260	CE2	3.76
		Wat86 (bridge to Tyr260 OH)	O	2.95
Ala4	CAX	Leu321	CD2	3.93
		Ala47	CB	3.47
Arg3	OAP	Wat12 (bridge to Cys261 N)	O	2.83
		Tyr260	CD2	3.86
Arg3	CAM	Phe133	CE1	3.26
		Phe133	CZ	3.60
		Ser91	OG	3.92
Arg3	CAW	Phe133	CE1	3.61
		Phe133	CZ	3.70
		Ser91	CB	3.76
		Ser91	OG	3.54
Arg3	CAR	Cys261	O	3.81
		Phe133	CZ	3.96
		Ile305	CG1	3.82
		Ile305	CD1	3.90
Arg3	CAQ	Cys261	O	3.65
		Phe263	CE1	3.77
		Ile305	CG1	3.80
		Ser49	O	3.27
		Ser91	O	3.60
Arg3	NAV	Phe263	CE1	3.61
		Phe263	CZ	3.97
		Ser49	O	3.98
		Ser91	C	3.86
		Ser91	O	2.74
Arg3	CAU	Phe133	O	3.94
		Phe133	CE2	3.70
		Phe263	CE1	3.44
		Phe263	CZ	3.48
		Ser91	O	3.67
Arg3	NAT	Cys261	C	3.87
		Cys261	O	2.98
		Cys261	CB	3.70
		Wat33 (bridge to Ser218 O, Ser175 O)	O	2.77
		Phe133	CE2	3.85
		Phe263	CD1	3.94
		Phe263	CE1	3.40
		Phe263	CZ	3.68

Arg3	NAS	Wat45 (bridge to Ser91 O)	O	2.92
		Phe133	C	3.93
		Phe133	O	2.97
		Phe133	CE2	3.77
		Cys134	CB	3.83
		Ser175	O	3.93
		Phe263	CZ	3.62
		Ser91	O	3.77
Arg3	NAO	Phe133	CD1	3.98
		Phe133	CE1	3.24
		Ser91	OG	3.06
Ala2	CAJ	Phe133	CE1	3.79
Ala2	CAI	Asp107	OD1	3.74
Ala2	CAH	Phe133	CD1	3.92
		Phe133	CE1	3.98
		Tyr131	CD1	3.75
		Asp107	OD1	3.49
Ala2	NAK	Ser91	OG	3.21
		Asp107	CG	3.34
		Asp107	OD1	2.81
		Asp107	OD2	3.09
Ser1	C	Ser91	OG	3.79
		Asp107	CG	3.81
		Asp107	OD1	3.66
		Asp107	OD2	3.20
Ser1	CA	Ser91	OG	3.89
		Gly89	C	3.79
		Gly89	CA	3.93
		Asp107	CG	3.71
		Asp107	OD1	3.73
		Asp107	OD2	3.20
Ser1	N	Gly89	C	3.88
		Gly89	CA	3.46
		Asp107	CG	3.93
		Asp107	OD2	3.09
		Ala65	CA	3.89
		Ala65	CB	3.85
Ser1	CB	Ser91	OG	3.92
		Ile90	C	3.87
		Ile90	O	3.47
		Ala65	CA	3.69
		Ala65	CB	3.82
Ser1	OG	Ser49	CB	3.68
		Ser91	CA	3.49
		Ser91	CB	3.70
		Ser91	OG	2.87
		Ile90	C	3.78
		Ile90	O	3.40
		Ser91	N	3.86

H-bond cut off < 3.5 Å, Van der Waals: 3.6-4.0 Å.

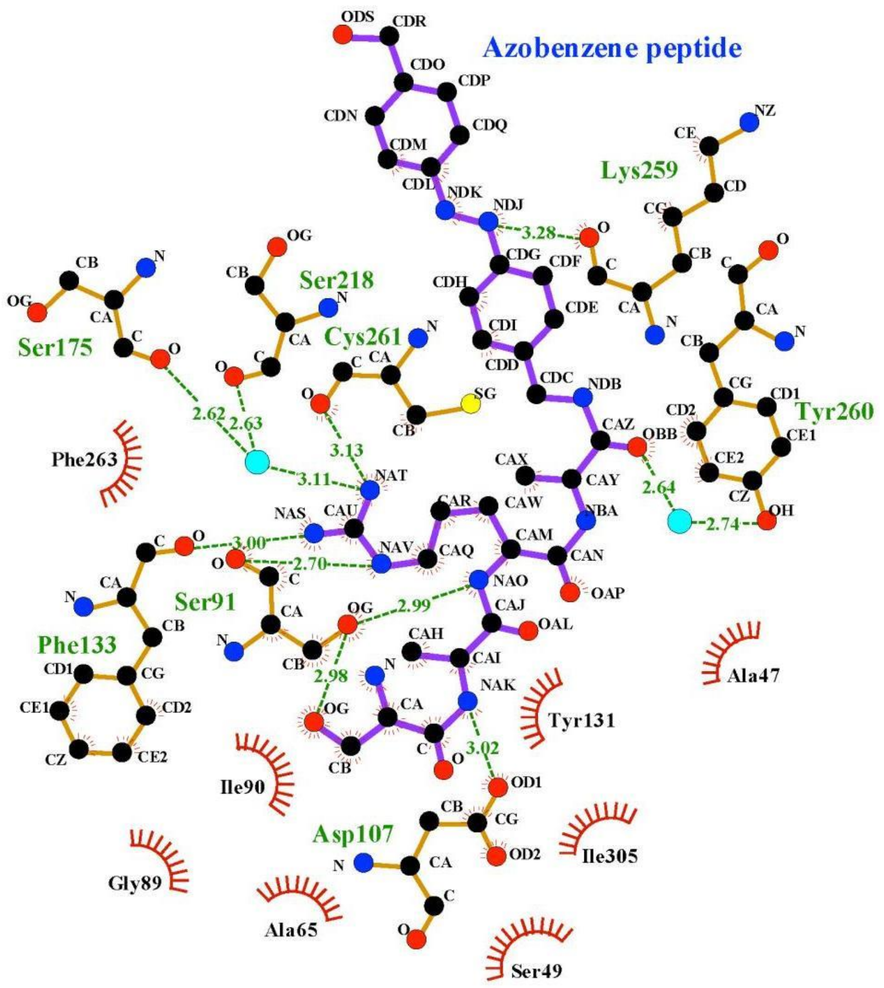

Figure S71. Schematic representation of the key interactions between the AMPB-peptide **7** in the state II and WDR5Δ23. Generated with LIGPLOT.[24]

Table S8. Interactions between the AMPB-peptide **7** in the state II and WDR5Δ23. Interactions marked in red, are strong H-bonds.

peptide		Hydrogen-bond partner		
Residue	Atom	WDR5 residue	Atom	Distance (Å)
AMPB5	CDM	Lys259	O	3.63
AMPB5	CDL	Lys259	O	3.98
		Lys259	CG	3.67
AMPB5	NDK	Lys259	O	3.91
		Lys259	CG	3.35
		Lys259	CE	3.69
AMPB5	NDJ	Lys259	O	3.28
			CG	3.54
AMPB5	CDH	Tyr260	CE2	3.53
		Tyr260	CD2	3.72
AMPB5	CDI	Tyr260	CE2	3.49
		Tyr260	CD2	3.72
Ala4	OBB	Tyr260	CE2	3.65
		Wat71 (bridge to	O	2.64

		Tyr260 OH)		
Ala4	CAX	Ala47	CB	3.82
Arg3	OAP	Tyr260	CD2	3.77
Arg3	CAM	Phe133	CE1	3.42
		Phe133	CZ	3.74
		Ser91	OG	3.87
Arg3	CAW	Phe133	CE1	3.57
		Phe133	CZ	3.60
		Ser91	CB	3.84
		Ser91	OG	3.50
Arg3	CAR	Cys261	O	3.99
		Ile305	CG1	3.78
		Ile305	CD1	3.71
Arg3	CAQ	Cys261	O	3.78
		Phe263	CE1	3.82
		Ile305	CG1	3.68
		Ser49	O	3.33
		Ser91	O	3.66
Arg3	NAV	Phe263	CE1	3.69
		Phe263	CZ	3.99
		Ser49	O	3.99
		Ser91	C	3.75
		Ser91	O	2.70
Arg3	CAU	Phe133	CE2	3.63
		Phe263	CE1	3.50
		Phe263	CZ	3.47
		Ser91	O	3.54
Arg3	NAT	Cys261	O	3.13
		Cys261	CB	3.73
		Wat27 (bridge to Ser218 O, Ser175 O)	O	3.11
		Phe133	CE2	3.65
		Phe263	CE1	3.48
		Phe263	CZ	3.63
Arg3	NAS	Asp92	OD2	3.92
		Phe133	C	3.91
		Phe133	O	3.00
		Phe133	CE2	3.60
		Phe133	CD2	3.62
		Phe263	CZ	3.58
		Ser91	O	3.56
Arg3	NAO	Phe133	CE1	3.43
		Ser91	OG	2.99
Ala2	CAJ	Ser91	OG	3.97
Ala2	CAI	Asp107	OD1	3.82
		Asp107	OD2	3.77
Ala2	CAH	Tyr131	CD1	3.78
		Asp107	OD1	3.45
Ala2	NAK	Ser91	OG	3.26
		Asp107	CG	3.32

		Asp107	OD1	3.02
		Asp107	OD2	2.83
Ser1	C	Ser91	OG	3.77
		Asp107	CG	3.95
		Asp107	OD1	3.95
		Asp107	OD2	3.15
Ser1	CA	Ser91	OG	3.87
		Asp107	CG	3.81
		Asp107	OD1	3.97
		Asp107	OD2	3.10
Ser1	N	Gly89	CA	3.69
		Asp107	OD2	3.13
		Ala65	CA	3.95
		Ala65	CB	3.80
Ser1	CB	Ser91	OG	3.87
		Ile90	N	3.94
		Ile90	C	3.63
		Ile90	O	3.07
		Ala65	CA	3.84
		Ala65	CB	3.93
Ser1	OG	Ser49	CB	3.45
		Ser91	CA	3.41
		Ser91	CB	3.73
		Ser91	OG	2.98
		Ile90	C	3.66
		Ile90	O	3.10
		Ser91	N	3.81

H-bond cut off < 3.5 Å, Van der Waals: 3.6-4.0 Å.

Evaluation of Stability against GSH-reduction of Peptides

To test the stability of the AMPB-containing peptides **16** and **17** against reduction by glutathione (GSH), the peptides were incubated with GSH for 4 days.

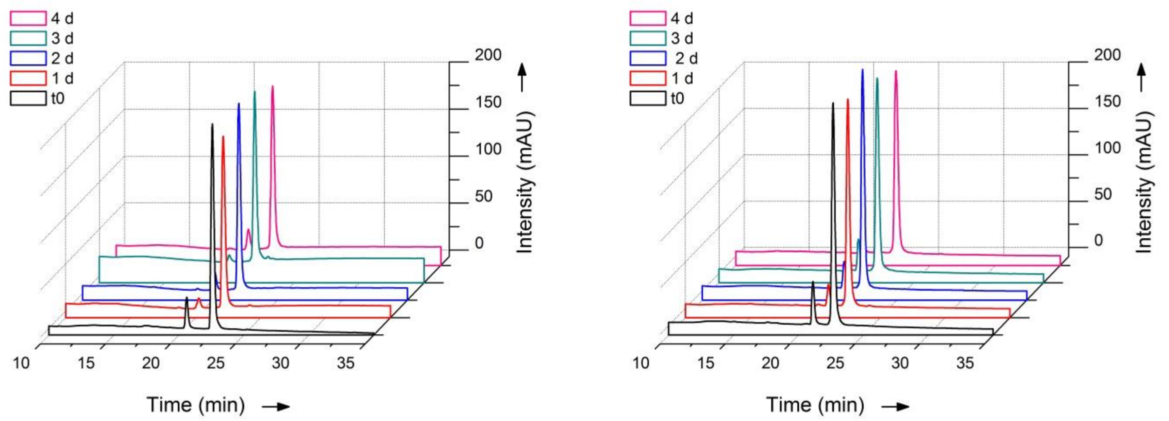

Figure S72. Left: HPLC chromatograms of peptides, incubated with 10 mM GSH, recorded after different time periods. Left: peptide **16**. Right: peptide **17**.

Therefore, 56 μL of a stock solution of GSH (0.25 M) and 1344 μL of a stock solution of peptides **16** and **17** (10 μM) in 0.1 M phosphate buffer pH = 7 were mixed giving final concentrations of 10 mM of GSH and 9.6 μM of the peptides, respectively. After different time periods, HPLC chromatograms of these mixtures were recorded (gradient: 5-40% B) (Figure S72).

In Vitro Methyl Transferase Assay with MLL1 Core Complex

Protein Expression

Full length constructs of both, RbBP5 (residues 1–538) and Ash2L (residues 1–635), were used for expression. Truncated WDR5 for robust HMT activity of the core complex, were used. MLL1, WDR5, RbBP5, and ASH2L were expressed as His-SUMO fusions from the pET28A-SUMO vector in BL21 DE3 pLyss codon (+) cells at 16 °C overnight. Expression was started with 0.1 mM IPTG in the mid-log phase of bacterial growth. For each protein, cells were harvested and the protein was purified by the His tag on Ni-NTA resin (Qiagen). The SUMO tag was removed from RbBP5, ASH2L, and MLL1 proteins by incubation with the ULP1 protease at 4 °C overnight. The protease and cleaved SUMO-His tag were collected by batch binding with the Ni-NTA resin for 1 h.

In Vitro Histone Methyltransferase (HMT) Assay

The HMT assay was performed, as previously described[25] with slight modifications, in 50 mM Tris pH 8.0, 50 mM NaCl, 1.0 mM DTT, 5 mM $MgCl_2$ at room temperature. Each reaction contained 1.5 μM of the cofactor ^{3}H-S-adenosylmethionine (Perkin-Elmer). $H3_{1\text{-}21}$ peptide was used as the substrate at 250 μM. Both, the *trans* and the *cis* (*cis*: previously irradiated at 366 nm for 1 h) isomer, of peptide **7** were added at concentrations ranging from 0.705 nM to 70.5 μM and incubated with the preassembled MLL1 complex at a final concentration of 0.3 μM for 10 min on ice. Reactions were initiated by addition of the H3/SAM and allowed to proceed for 60 min before preparing scintillation counting. To count samples, reactions were spotted on separate squares of P81 filter paper (Millipore) and precipitated by submerging in freshly prepared 50 mM sodium bicarbonate buffer pH = 9.0. After washing and drying, the samples were vortexed in Ultima Gold scintillation fluid and counted.

Proliferation Assay

Previously to the cell viability assays, it was tested for how long the cells could be irradiated with the custom made 6-well LED array (430 nm), without damaging. Therefore, MLL-AF9-transduced mouse bone marrow cells were irradiated for different time periods (Figure S73).

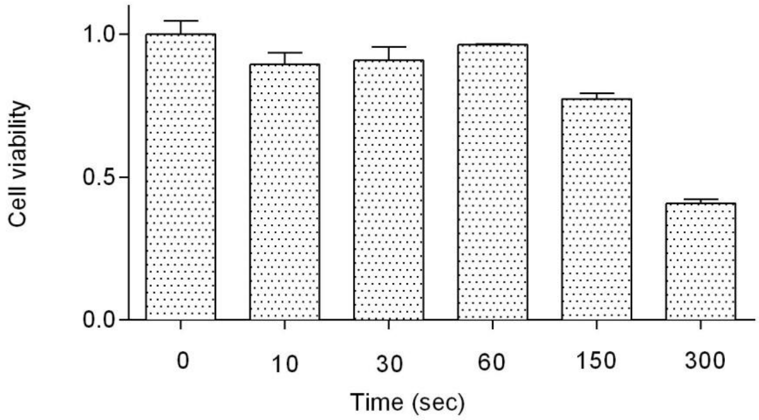

Figure S73. Cell viability after MLL-AF9-transduced mouse bone marrow cells were irradiated at 430 nm with the custom made 6-well LED array for different time periods.

MLL-AF9-transduced mouse bone marrow was cultured in IMDM medium + 15% FBS and supplemented with 10 ng/mL interleukin-3 (IL-3) at each passage, every other day.

Both, the *trans* and the *cis* (*cis*: previously irradiated at 366 nm for 4 h at 100 mM stock in DMSO) isomers, of peptides **7**, **16**, **17**, as well as the peptides **18** and **19** were diluted from stock to culture media containing a 0.1% DMSO final concentration. For viability assays, cells were cultured at 1 × 10^4/mL in 6-well plates with 2.5% FBS and treated with each peptide at 1.0, 1.6, 2.5, 5.0, 10, 25, 50 and 100 µM for 24 h and then added FBS up to 15% for 72 h and DMSO as control. For the *cis*-*trans* group, after 48 h treatment with *cis* peptides, the plate was irradiated at 430 nM for 90 sec and continued to culture for 2 days in the dark. Viability was determined using the CellTiter-Glo Kit (Promega) according to the manufacturer's directions. Luminescence was monitored on a Molecular Devices plate reader.

Real Time Quantitative PCR Analysis of Deptor Gene

Murine MLL1-AF9 transformed bone marrow cells were obtained by transducing normal murine bone marrow cells with MLL1-AF9 oncogene according to the procedures described by Tan et al.[26] Both, the *trans* and the *cis* (*cis*: previously irradiated at 366 nm for 4 h for 100 mM stock) isomers, of peptides **7**, **16**, **17**, as well as the peptides **18** and **19** were dissolved in DMSO.

The transformed cells were treated with different concentrations of the respective peptide (1.0, 5.0, 10 μM) and Mock (0.1% DMSO), giving a final concentration of 0.1% DMSO in all the samples. Total RNA was isolated from MLL1-AF9 transduced mouse bone marrow cells after 4 day treatment, using Trizol (Invitrogen) and the RNEASY kit (Qiagen) according to the protocol described earlier.[27] The cDNA was generated using random priming with the SuperScript III kit (Invitrogen). Real-time PCR amplifications of Deptor and GAPDH genes were carried out with primers specific for each gene in the presence of SYBR dye. Relative quantification of each gene transcript was carried out as described previously.[28] The results are presented in Fig8 in the manuscript as relative expression to Mock treatment after normalizing to an internal loading control (e.g. GAPDH).

References

[1] W. J. Fang, T. Yakovleva, J. V. Aldrich, *Biopolymers* **2011**, *96*, 715–722.

[2] W. S. Hancock, J. E. Battersby, *Anal. Biochem.* **1976**, *71*, 260–264.

[3] N. Thieriet, J. Alsina, E. Giralt, F. Guib??, F. Albericio, *Tetrahedron Lett.* **1997**, *38*, 7275–7278.

[4] P. Grieco, P. M. Gitu, V. J. Hruby, *J. Pept. Res.* **2001**, *57*, 250–256.

[5] C. Renner, J. Cramer, R. Behrendt, L. Moroder, *Biopolymers* **2000**, *54*, 489–500.

[6] M. Löweneck, A. G. Milbradt, C. Root, H. Satzger, W. Zinth, L. Moroder, C. Renner, *Biophys. J.* **2006**, *90*, 2099–2108.

[7] H. Rau, *Stud. Org. Chem. Photochroism, Mol. Syst.* **1990**, *40*, 165–192.

[8] D. A. James, D. C. Burns, G. A. Woolley, *Protein Eng.* **2001**, *14*, 983–991.

[9] S.-L. Dong, M. Löweneck, T. E. Schrader, W. J. Schreier, W. Zinth, L. Moroder, C. Renner, *Chem. Eur. J.* **2006**, *12*, 1114–1120.

[10] B. Priewisch, K. Rück-Braun, *J. Org. Chem.* **2005**, *70*, 2350–2352.

[11] R. Sjöback, J. Nygren, M. Kubista, *Spectrochim. Acta Part A Mol. Biomol. Spectrosc.* **1995**, *51*, L7–L21.

[12] Invitrogen Corporation, *Technical Resource Guide, Fluorescence Polarization, 4th Edition*, Madison, **2005**.

[13] Z. Nikolovska-Coleska, R. Wang, X. Fang, H. Pan, Y. Tomita, P. Li, P. P. Roller, K. Krajewski, N. G. Saito, J. a. Stuckey, et al., *Anal. Biochem.* **2004**, *332*, 261–273.

[14] W. Kabsch, *Acta Crystallogr. Sect. D Biol. Crystallogr.* **2010**, *66*, 125–132.

[15] M. D. Winn, C. C. Ballard, K. D. Cowtan, E. J. Dodson, P. Emsley, P. R. Evans, R. M. Keegan, E. B. Krissinel, A. G. W. Leslie, A. McCoy, et al., *Acta Crystallogr. Sect. D Biol. Crystallogr.* **2011**, *67*, 235–242.

[16] A. J. McCoy, R. W. Grosse-Kunstleve, P. D. Adams, M. D. Winn, L. C. Storoni, R. J. Read, *J. Appl. Crystallogr.* **2007**, *40*, 658–674.

[17] P. D. Adams, P. V. Afonine, G. Bunkóczi, V. B. Chen, I. W. Davis, N. Echols, J. J. Headd, L. W. Hung, G. J. Kapral, R. W. Grosse-Kunstleve, et al., *Acta Crystallogr. Sect. D Biol. Crystallogr.* **2010**, *66*, 213–221.

[18] P. Emsley, K. Cowtan, *Acta Crystallogr. Sect. D Biol. Crystallogr.* **2004**, *60*, 2126–2132.

[19] A. W. Schüttelkopf, D. M. F. Van Aalten, *Acta Crystallogr. Sect. D Biol. Crystallogr.* **2004**, *60*, 1355–1363.

[20] V. B. Chen, W. B. Arendall, J. J. Headd, D. A. Keedy, R. M. Immormino, G. J. Kapral, L. W. Murray, J. S. Richardson, D. C. Richardson, *Acta Crystallogr. Sect. D Biol. Crystallogr.* **2010**, *66*, 12–21.

[21] W. L. DeLano, *The PyMOL User's Manual*, DeLano Scientific, **2002**.

[22] A. Patel, V. Dharmarajan, M. S. Cosgrove, *J. Biol. Chem.* **2008**, *283*, 32158–32161.

[23] J. J. Song, R. E. Kingston, *J. Biol. Chem.* **2008**, *283*, 35258–35264.

[24] A. C. Wallace, R. A. Laskowski, J. M. Thornton, *Protein Eng.* **1995**, *8*, 127–134.

[25] F. Cao, E. C. Townsend, H. Karatas, J. Xu, L. Li, S. Lee, L. Liu, Y. Chen, P. Ouillette, J. Zhu, et al., *Mol. Cell* **2014**, *53*, 247–261.

[26] J. Tan, M. Jones, H. Koseki, M. Nakayama, A. G. Muntean, I. Maillard, J. L. Hess, *Cancer Cell* **2011**, *20*, 563–575.

[27] X. Li, L. Li, R. Pandey, J. S. Byun, K. Gardner, Z. Qin, Y. Dou, *Cell Stem Cell* **2012**, *11*, 163–178.

[28] Y. Dou, T. A. Milne, A. J. Ruthenburg, S. Lee, J. W. Lee, G. L. Verdine, C. D. Allis, R. G. Roeder, *Nat. Struct. Mol. Biol.* **2006**, *13*, 713–719.
